# Deep origins, distinct adaptations, and species-level status indicated for a glacial relict seal

**DOI:** 10.1073/pnas.2503368122

**Published:** 2025-06-10

**Authors:** Ari Löytynoja, Jaakko Pohjoismäki, Mia Valtonen, Juha Laakkonen, Wataru Morita, Mervi Kunnasranta, Risto Väinölä, Morten Tange Olsen, Petri Auvinen, Jukka Jernvall

**Affiliations:** ^a^Organismal and Evolutionary Biology Research Programme, Faculty of Biological and Environmental Sciences, University of Helsinki, Helsinki 00014, Finland; ^b^Department of Environmental and Biological Sciences, University of Eastern Finland, Joensuu 80101, Finland; ^c^Natural Resources Institute Finland (Luke), Helsinki 00791, Finland; ^d^Division of Veterinary Anatomy and Developmental Biology, Department of Veterinary Biosciences, Faculty of Veterinary Medicine, University of Helsinki, Helsinki 00014, Finland; ^e^Division of Anthropology, Department of Paleontology and Anthropology, National Museum of Nature and Science, Tsukuba Ibaraki, Japan; ^f^Natural Resources Institute Finland (Luke), Joensuu 80100, Finland; ^g^Finnish Museum of Natural History, University of Helsinki, Helsinki 00014, Finland; ^h^Section of Molecular Ecology and Evolution, Globe Institute, University of Copenhagen, Copenhagen, Denmark; ^i^Institute of Biotechnology, Helsinki Institute of Life Science, University of Helsinki, Helsinki 00014, Finland; ^j^Department of Geosciences and Geography, University of Helsinki, Helsinki 00014, Finland

**Keywords:** mammal, genomic analysis, phenotypic analysis, freshwater pinniped, Lake Saimaa

## Abstract

Isolated populations of postglacial relicts are known from many regions and are typically found on mountains for terrestrial species and in lakes for aquatic species. Among the few aquatic mammalian relicts, the Saimaa ringed seal (*Pusa hispida saimensis*) has been landlocked in Lake Saimaa, Finland, for the last 10,000 y. Saimaa ringed seals show genetic, behavioral, and morphological differences from the other ringed seal subspecies, but the extent these differences stem from the end of the last glacial period remains unclear. Here, we demonstrate with comprehensive sampling and state-of-the-art genomic methods that the Saimaa ringed seals are much older than the lake they inhabit, having formed a separate evolutionary branch for at least 60,000 y. This deep evolutionary origin of the Saimaa ringed seals is further underscored by anatomical differences, including our ecomorphological analyses revealing adaptively distinct features in their dentition and tongue. Overall, glacial relicts may harbor a richer selection of evolutionary history than might be expected from their recent isolation history alone.

## Significance

Isolated populations of postglacial relicts are typically found on mountains for terrestrial species, and in lakes for aquatic species. Most of the isolated populations have formed after the end of the last glacial period. One such aquatic population is the Saimaa ringed seal living landlocked in Lake Saimaa, Finland. We found using genomic analyses that the evolutionary lineage leading to Saimaa ringed seals is much older than the lake itself. Morphological data on dentitions also showed Saimaa ringed seals to be adaptively distinct. Glacial relicts can harbor higher species richness than previously thought, emphasizing the need for continued conservation efforts in the face of climate change and human threats.

**Fig. 1.**
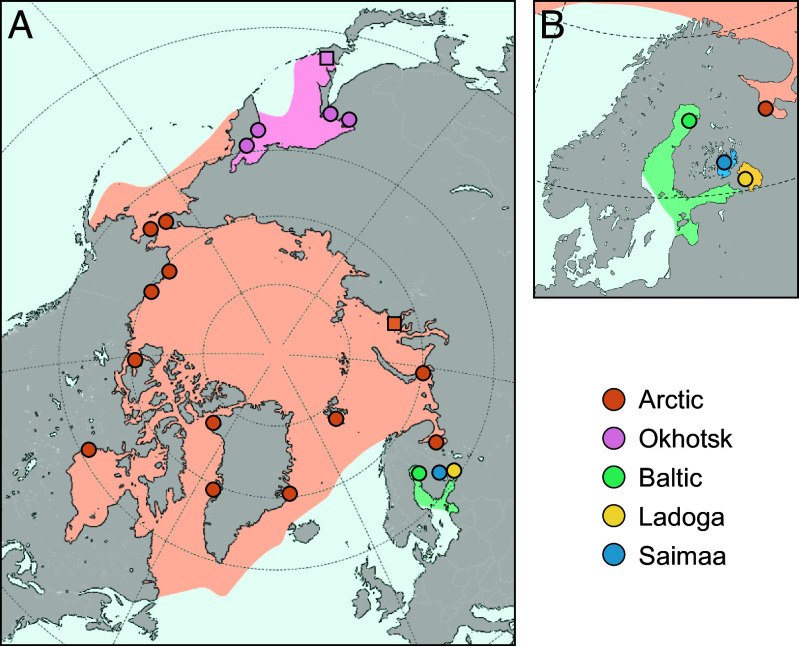
The distribution and sampling of the studied ringed seals and their distribution ranges. (*A*) The nominal subspecies of the Arctic ringed seal (*P. h. *hispida**) has circumpolar distribution while the four other subspecies are geographically restricted. (*B*) The ringed seals living in Fennoscandia are made of landlocked populations in Lake Saimaa and Lake Ladoga, and the Baltic Sea population. Phenotypic sampling from museum collections roughly matches those of the genetic data, except for Arctic samples from the Kara Sea (square) substituting for the Pechora Sea, and Sea of Okhotsk samples being from Hokkaido or nearby islands (square).

Ringed seals (*Pusa hispida*) are the most common Arctic pinnipeds, presently found throughout all seasonally ice-covered northern seas and two freshwater lakes (Fig. 1*A*). They are highly ice-associated, adapted to maintaining breathing holes and constructing subnivean lairs for parturition, nursing, and resting (1, 2). The nominate subspecies, *P. hispida hispida* (Schreber, 1775) (hereafter the “Arctic ringed seal”) is the most widespread, inhabiting the circumpolar Arctic Ocean. Three subspecies are present as distinct populations in Fennoscandia (Fig. 1*B*), the Baltic ringed seal *P. h. botnica* (Gmelin, 1788), the Ladoga ringed seal *P. h. ladogensis* (Nordqvist, 1899), and the Saimaa ringed seal *P. h. saimensis* (Nordqvist, 1899). The presence of these three Fennoscandian subspecies has been explained by the isolation of the ringed seals into the Baltic Sea basin after the last glacial period with further entrapment of seals into Lake Ladoga and Lake Saimaa (3). Lake Saimaa is a freshwater lake with a surface area of 4,400 km^2^ and houses a population of close to 500 endemic ringed seals, making these one of the most endangered pinnipeds in the world (4, 5). Currently, the population is slowly growing due to active conservation efforts, but the Saimaa ringed seal is still threatened by bycatch mortality and climate change driven habitat change (6).

The morphological and ecological differences between the Saimaa ringed seal and the other subspecies have been long known (7–12), historically assumed to derive from the Holocene postglacial isolation of the Saimaa population from the Baltic Sea population. However, genetic analyses of mitochondrial DNA (mtDNA) haplotypes (13–17), microsatellite loci (17–19), and genome-wide variation (20, 21) have revealed greater genetic differences between Saimaa and other populations than can be explained by postglacial drift and recent adaptation.

In this study, we explore more broadly the genomic differences between the Saimaa and other ringed seals by including the previously unsampled Arctic ringed seals in northern Eurasia, as well as the Okhotsk ringed seal *P. h. ochotensis* (Pallas, 1811), an Asian Pacific subspecies (Fig. 1 and *SI Appendix*, Tables S1 and S2). In addition to mitochondrial and nuclear genomes, we examine feeding morphology differences to address the magnitude of ecological differentiation among the subspecies. We show that even with circumpolar sampling of Arctic ringed seal populations, the Saimaa ringed seal retains its genetic uniqueness. Based on the genomic divergence from the other ringed seals, together with specialized morphological features linked to feeding ecology, we argue that it is justified to treat the Saimaa ringed seals as bona fide species, *Pusa saimensis* stat. nov. and not as a geographic form or a subspecies of the Arctic ringed seal.

## Results

### Genetic Origins and Differentiation of the Saimaa Ringed Seal.

Our phylogenetic analysis of mtDNA data, representing the first truly global sampling of mitochondrial diversity in ringed seals, confirms earlier findings (13, 15, 16, 19) and shows Saimaa forming a tight cluster with closely related haplotypes present but rare among the Baltic and Arctic ringed seals (*SI Appendix*, Fig. S1). The wider sampling from the eastern hemisphere fails to establish connections between Saimaa and other populations. Similarly to Ladoga and Baltic ringed seals, the haplotypes of the sampled Okhotsk individuals are spread into multiple distinct lineages of the phylogenetic tree (*SI Appendix*, Fig. S1). In contrast, despite being embedded within the global mtDNA variation, the Saimaa population appears to form the only monophyletic lineage of mtDNA haplotypes (15, 16).

**Fig. 2.**
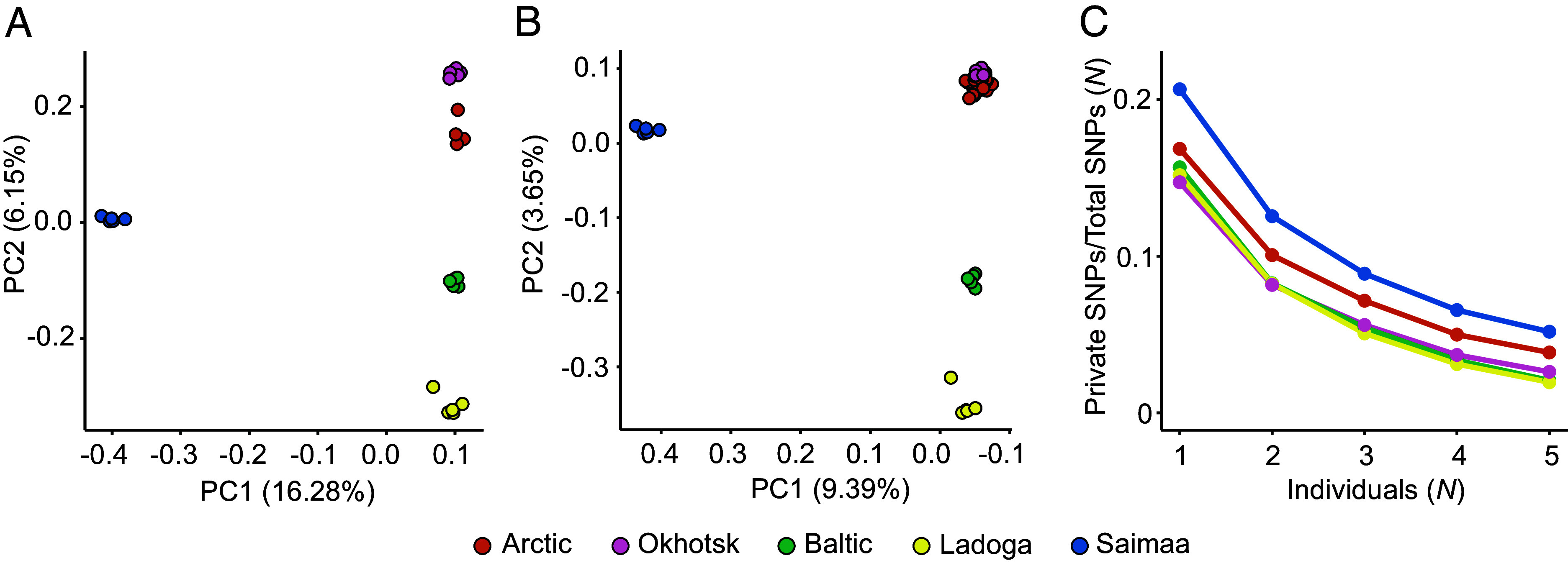
Genetic uniqueness of the Saimaa ringed seals. (*A*) Using five individuals from each population, the Saimaa ringed seals form a tightly defined and separate cluster along the PC1 in the PCA, while the genetic differentiation of the non-Saimaa seals creates a geographic continuum along the PC2: the Baltic are placed between the Ladoga and Arctic, and Okhotsk further away. The Arctic individual placed between the rest of the Arctic and Okhotsk is from the Bering Strait. (*B*) Comparable patterns are obtained using the full 46 individual data (*SI Appendix*, Table S1), although the uneven sampling hides the separation between Arctic and Okhotsk. The *x*-axis is reversed to visually match (*A*). (*C*) With MAF 0.05 filtering, Saimaa has the highest proportion of population-specific derived alleles in samples of 1 to 5 individuals per subspecies.

Compared to the nonrecombining mtDNA haplotypes, the nuclear genome is expected to provide a more nuanced view into population histories. We started by measuring the overall differences between the nuclear genomes and performed a principal component analysis (PCA) on 1.808 million SNPs from five individuals per population. The Saimaa sample forms a tight cluster and is separated from all the others, including the Okhotsk ringed seal, by PC1, whereas the other populations are separated by PC2 (Fig. 2*A*). The full 46 individual dataset (*SI Appendix*, Table S1) produces a largely similar division between the populations but places the Arctic and Okhotsk samples closer to each other (Fig. 2*B*), possibly as an artifact of uneven sampling (22). We continued by computing the derived allele statistics and found that the Saimaa ringed seals retain a high representation of population-specific variants, a pattern preserved when multiple individuals are compared (Fig. 2*C*). The relative magnitude of the private variants in Saimaa makes it highly implausible that this variation has resulted from drift during the 10 kya, or roughly 1,000-generation entrapment in the lake. Finally, we generated phylogenetic trees from the nuclear genomes. These show highly complex patterns where different parts of the genome produce different trees (*SI Appendix*, Fig. S2), an outcome to be expected due to incomplete lineage sorting and admixture among closely related taxa (23, 24). Yet, 95.4% of the nuclear DNA regions support the monophyly of Saimaa while no other node is supported by a majority (50%) of the trees (*SI Appendix*, Fig. S2). A recent comprehensive mtDNA analysis of ringed seals suggested several Fennoscandian colonization events (16), potentially explaining some of the admixture events causing the complex genomic patterns. Overall, although Saimaa ringed seals appear distinct and monophyletic, the history of ringed seals is complex and cannot be displayed as a simple tree.

**Fig. 3.**
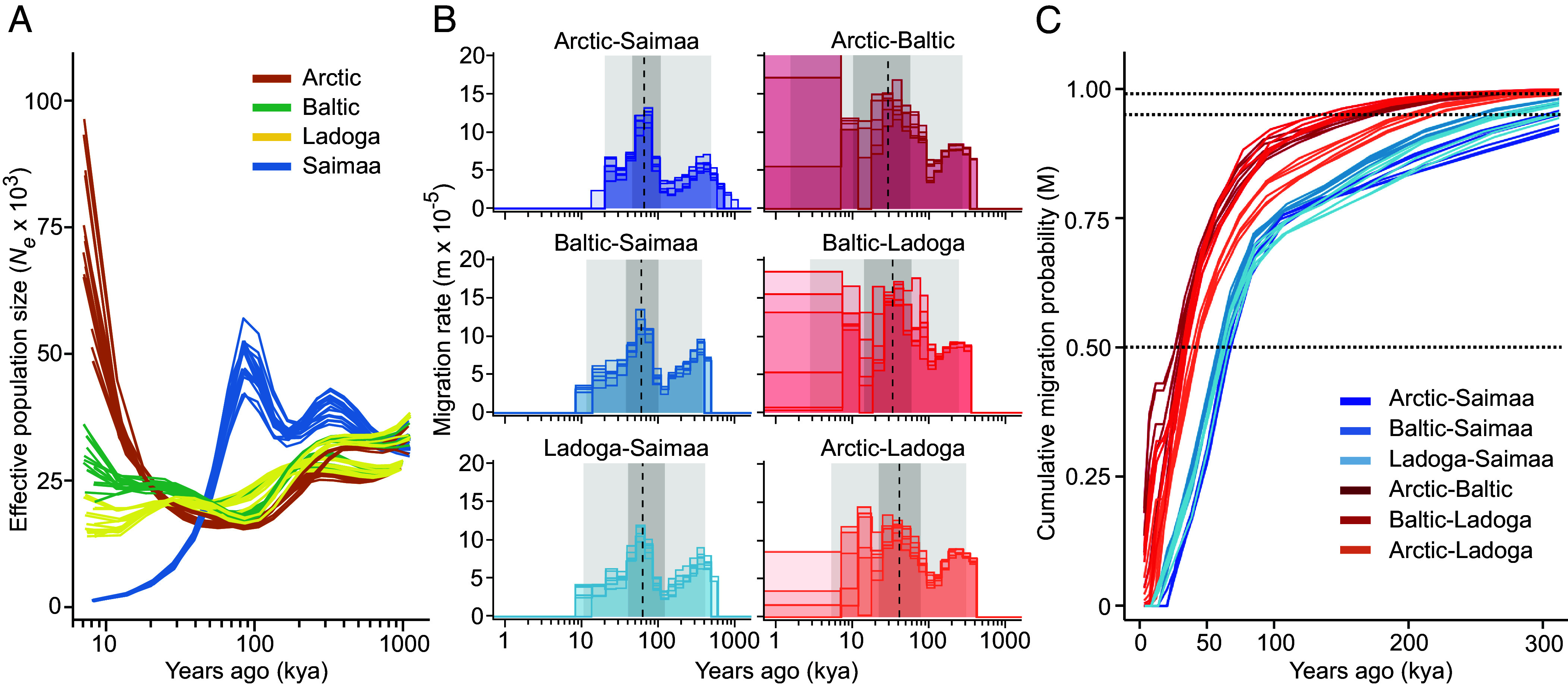
Estimated past effective population sizes and migration rates reveal a deep history for Saimaa ringed seals. (*A*) Estimates of past effective population sizes (*N_e_*) for the three Fennoscandian and Arctic ringed seals (the distant Sea of Okhotsk is not included in these analyses) as computed from the full genome data. (*B*) Estimates of migration rates over time between pairs of individuals from different populations. The rates are cut at the point where the cumulative migration probability reaches 0.999. Background shadings indicate the average cumulative probability of 0.01, 0.25, 0.75, and 0.99, and dashed lines that of 0.50. (*C*) Cumulative migration probability between pairs of individuals from different populations. The dotted lines indicate the thresholds M_50_, M_95_, and M_99_, the x-coordinates of intersections defining the age of each pair reaching the respective level. Five individuals per population were included and each line represents a comparison of individuals from two different populations. Mutation rate (μ) and generation time were 1.826e-8 and 10 y, respectively.

To better understand the population histories, we focused on the divergence times among the three Fennoscandian populations (Baltic, Ladoga, Saimaa) in reference to the Arctic population using MSMC–IM (25), a demographic inference method providing time-dependent estimates of population-specific *N_e_* as well as gene flow. Even with this improved modeling compared to a previous analysis (20), the inferred *N_e_* trajectories for the Saimaa population remain distinctly different from those of other populations throughout most of the late-glacial period. Notably, the Saimaa population shows a dramatic decline that appears to begin before the seals could enter the present lake basin (Fig. 3*A*). In contrast, the other populations follow similar trajectories in the deeper time, prior to their postulated split. The estimates of migration rate over time (Fig. 3*B*) demonstrate the recent strict isolation of the Saimaa population but also suggest the Saimaa lineage to have been in contact with the Baltic and Ladoga population around 8 to 10 kya and the exchange being at its greatest around 50 to 80 kya (Fig. 3*B*). The gene flow among the three Fennoscandian populations between 8 to 10 kya provides an explanation for the presence of Saimaa-associated mtDNA haplotypes and microsatellite alleles – although at low frequency –in the Baltic population (13, 17–19), as well as for the small proportion of the genome not supporting Saimaa monophyly (*SI Appendix*, Fig. S2).

To further date the roots of the Saimaa lineage, we computed M, the cumulative migration probability over time, between each population pair (Fig. 3*C*). These confirm the clearly distinct accumulation of gene flow between Saimaa and the three other populations, while the separate trajectories for the Arctic-Ladoga pairs support multiple waves of gene flow from the Atlantic to the Baltic region, some of that not reaching Ladoga. As a proxy for the population split times (25), we estimated the times for M reaching 50% (M_50_) as well as the more stringent thresholds of 95% (M_95_) and 99% (M_99_) (Table 1). With the applied parameters of mutation rate (*μ*) and generation time (*g*), M_50_ for Saimaa varies between 60.2 kya (with Baltic) and 65.8 kya (with Arctic). These values are about twice as high as those among the three non-Saimaa populations, varying from 29.2 kya to 41.7 kya (Table 1). The much greater estimates of M95 and M99 for comparisons including Saimaa individuals indicate that parts of the Saimaa genome have even deeper ancestry (Table 1). It is also notable that the M cut-off values for the Saimaa-Ladoga pairs are deeper in the past than they are for the Saimaa-Baltic pairs, highlighting the distinct origins of the two lake populations (16).

**Table 1.** Average age (years ago) for cumulative migration probability reaching 50%, 95%, and 99% for different population pairs

	M_50_	SD_50_	M_95_	SD_95_	M_99_	SD_99_
Arctic-Saimaa	65,819	1,770	336,303	29,985	490,069	50,029
Baltic-Saimaa	60,220	1,692	261,615	10,421	372,045	14,322
Ladoga-Saimaa	63,152	2,033	294,702	21,567	413,519	32,510
Arctic-Baltic	29,199	2,874	169,737	5,719	272,638	6,849
Baltic-Ladoga	33,502	1,151	148,504	10,678	244,162	12,628
Arctic-Ladoga	41,718	1,369	209,608	8,912	310,000	14,762

SD = standard deviation.

An early isolation of the Saimaa lineage between 10 to 60 kya could be explained by a refugium in the glacial lakes either on the eastern or southern side of the Fennoscandian ice sheet. During 90 to 50 kya, large glacial lakes existed in the West Siberian Plains and White Sea Basin with water routes to the eastern edge of the Fennoscandian ice sheet (26–28). Similarly, a chain of ice lakes existed at the southeastern border of the glacier, in current-day western Russia and Belarus, with connections to the Caspian Sea and the Black Sea (29). After the latest glacial maximum, around 11.6 kya, the Baltic Ice Lake drained to the Atlantic, creating a brief phase of the brackish-water Yoldia Sea (30) followed by the re-enclosed Ancylus Lake (31). Migration of seals between the Atlantic and the Baltic Sea basin has been permanently possible since the opening of the connection through the Danish Straits 8.5 kya (3), and also detectable in the genetic data (32).

Taken together, the genomic evidence demonstrates that Saimaa ringed seals represent an evolutionary lineage with deep roots, clearly distinct from the contemporary Baltic and Ladoga ringed seals that were postglacially derived from the Arctic population. The approximately two-fold divergence time indicates that the ancestors of the Saimaa population might have become isolated in an ice-dammed lake system east or southeast of the continental ice sheet already during the build-up of the last Fennoscandian Ice sheet. Regardless of the precise geographic scenario, and used mutation rate and generation time parameters (*Materials and Methods*), the population size trajectory points to an even older independent history for the Saimaa lineage (Fig. 3). Along with this unique ancestry, the Saimaa lineage also carries ancestry derived from a postglacial admixture with Arctic ringed seals. Currently, the land-locked Saimaa ringed seals are effectively allopatric species and thus continuing their distinct evolutionary trajectory into the foreseeable future. This distinct evolutionary trajectory is not limited to the Saimaa ringed seals, as also their seal lice are genetically distinct from the other studied ringed seal lice (33).

### Phenotypic Differentiation of the Saimaa Ringed Seal Relating to Feeding Ecology.

Given the deep ancestry of the Saimaa lineage, next we examined phenotypic features that may relate to feeding ecology differences between marine and freshwater habitats. Previous analyses have demonstrated morphological features, especially related to the skull that differentiates Saimaa ringed seals from other ringed seals (7–9, 12, 16). A short postcanine tooth row has been reported to characterize the Saimaa ringed seal (12) and here we analyzed the dentition in greater detail. In the seals, the spacing of teeth, hence the length of the tooth row increases as the individual grows. Thus, as with other cranial and skeletal features, age-controlled sampling must be used for tooth row length. In contrast, individual tooth measurements are affected only by wear, rarely an issue with ringed seals as they lack exact occlusion and do not chew their food (but see ref. 34). Instead, the laterally compressed postcanines are used for biting and piercing of food, or retention of food in the oral cavity when the water is expelled (35–39).

**Fig. 4.**
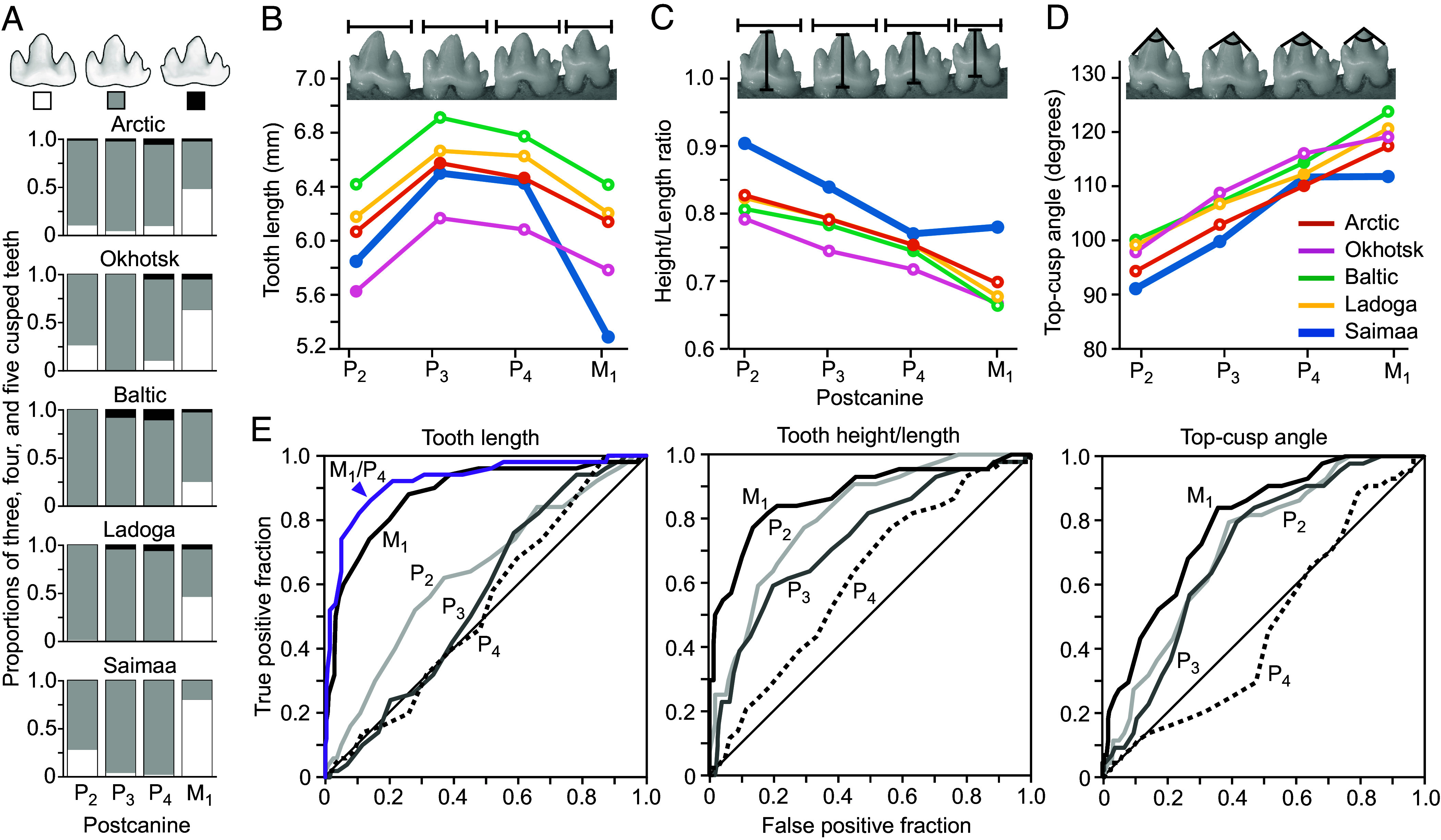
Saimaa ringed seals are distinct in their dental anatomy. (*A*) The lower postcanines of ringed seals have typically four cusps but can have also three or five cusps, the latter being absent in Saimaa. (*B*) Mean lengths of lower premolars P_2_ to P_4_ of Saimaa ringed seals are generally smaller than in Baltic and Ladoga, but only M_1_ is distinctly small compared to all the other populations. In the Saimaa dentition, the relative tooth heights (*C*), and the top-cusp angles measuring relative height of the lateral cusps (*D*), are the highest and smallest, respectively. These indicate shaper crown profiles in the Saimaa ringed seal, whereas the other populations retain the more characteristic ringed seal morphology irrespective of the size (*B*). Means differing from Saimaa with *P*-values below 0.05 are marked with open symbols in (*B*) to (*D*). For the *P* values, see *SI Appendix*, Table S5. (*E*) ROC curves showing the performance of different teeth and measures in the classification of Saimaa ringed seals, M_1_ and to a lesser degree P_2_ performing the best for all the measures. For the length, removing absolute size variation by calculating M_1_/P_4_, improves the ROC curve further (purple). The illustrated tooth row is from Saimaa (UEF821) and the tooth outlines in (*A*) depict P_4_’s from the Arctic.

First, we compared the sizes of the largest four lower postcanines, consisting of three premolars (P_2_, P_3_, P_4_), and one molar (M_1_). These relatively uniform teeth are characteristic to phocid species and in ringed seals have three to five sharp cusps (Fig. 4*A*). In our 326 specimen sample covering Arctic, Okhotsk, Baltic, Ladoga, and Saimaa (*SI Appendix*, Table S3), only the Saimaa sample lacks five cusped teeth (Fig. 4*A* and *SI Appendix,* Table S4). Examining continuous variables, the anteroposterior postcanine lengths show that the Saimaa P_2_, to P_4_, are intermediate between the longer Baltic, Ladoga, and Arctic, and the shorter Okhotsk specimens (Fig. 4*B* and *SI Appendix*, Tables S4 and S5). However, the last postcanine M_1_ is distinctly short in Saimaa, even shorter than the M_1_ of Okhotsk seals, the smallest of the studied ringed seals (*P* = 0.0000 to 0.0011, Fig. 4*B* and *SI Appendix*, Tables S4 and S5). The small size and reduced cusp number of M_1_ (Fig. 4 *A* and *B*) suggest reduction in posterior dentition. Indeed, examining Saimaa tooth heights reveals that especially their anterior postcanines (P_2_ and P_3_) are tall (*SI Appendix*, Table S4), and their overall height/length ratios, measuring relative height of the crown profile, are by far the highest among the populations (Fig. 4*C* and *SI Appendix*, Tables S4 and S5). Furthermore, the top-cusp angles, measuring relative height of the lateral cusps, are the smallest in Saimaa (Fig. 4*D* and *SI Appendix*, Tables S4 and S5), further indicating a sharper crown profile for the Saimaa ringed seal. This morphological pattern contrasts with that of the characteristic ringed seal morphology observed in the other population samples, irrespective of their tooth size.

To explore the diagnostic value of tooth measures in identifying Saimaa ringed seals from the whole sample, we computed the receiver operating characteristic (ROC) curves for each tooth (Fig. 4*E*). For the tooth length the areas under the ROC curves, which measure the performance, are 0.64, 0.56, 0.53, and 0.88 for P_2_, P_3_, P_4_, and M_1_, respectively (Fig. 4*E*). Because the P_4_ appears to perform closest to random classification (area is closest to 0.5 and the line follows the diagonal in Fig. 4*E*), we tested whether it could be used to remove overall size variation from M_1_ by calculating the M_1_/P_4_ ratio for each specimen. The M_1_/P_4_ ROC curve shows even better performance than the M_1_ length alone, enclosing 0.92 of the area (Fig. 4*E*). A cut-off of approximately 0.88 for the M_1_/P_4_ ratio is indicated by the ROC curve as a diagnostic threshold value in the identification of Saimaa ringed seal material. Importantly, this size ratio is not affected by the overall small size of Okhotsk seals and can also be used irrespective of age and sex, although females show better separation (*SI Appendix*, Fig. S3). In addition to tooth length, both height/length ratios and top-cusp angles of M_1_ provide diagnostic value, enclosing 0.87 and 0.79 of the ROC area, respectively (Fig. 4*E*).

**Fig. 5.**
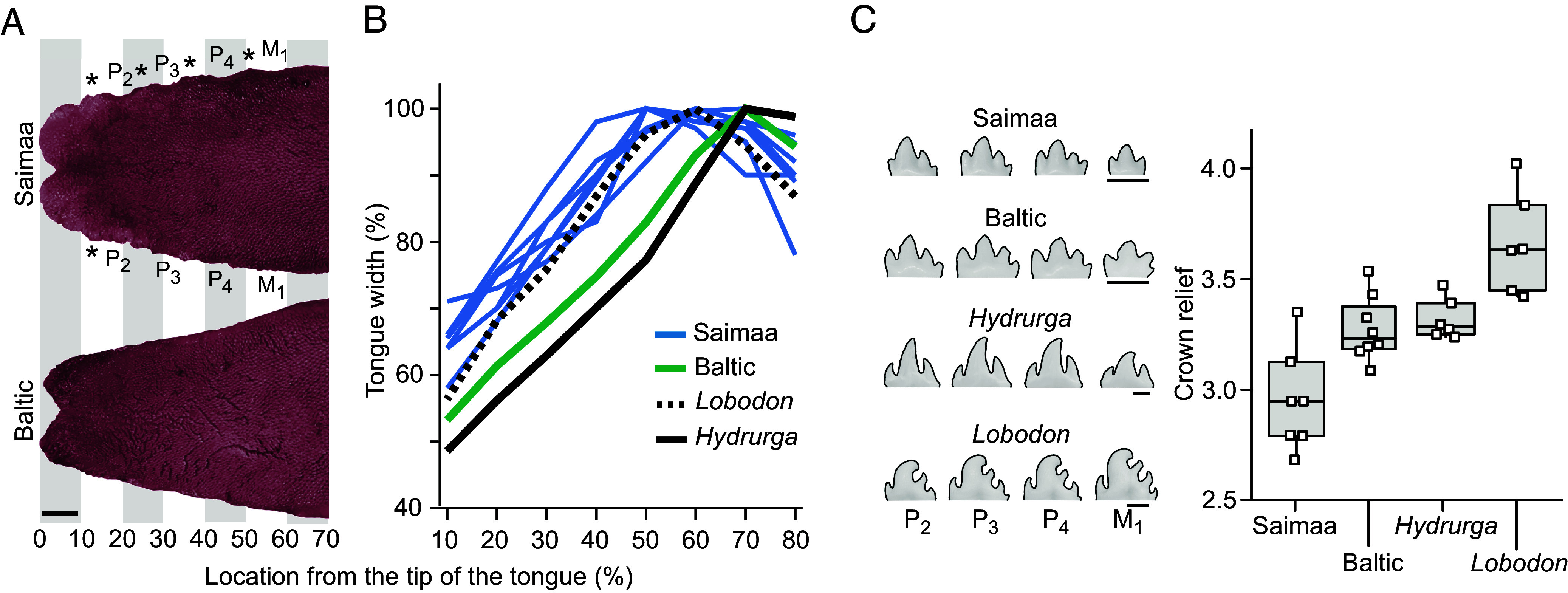
Tongue and dental morphology suggest increased suction but decreased filter-feeding niche for the Saimaa ringed seal. (*A*) Saimaa ringed seals have a broader tongue with a more rounded bifurcated tip, and intermolar elevations on the lateral side of the tongue (asterisks). (*B*) Comparison of tongue shapes with crabeater seal (*Lobodon*) and leopard seal (*Hydrurga*) tongues (*Material and methods*) shows that the Saimaa ringed seals have a more evenly broad tongue. (*C*) The cusp relief measuring the filtering capacity of teeth is lower in Saimaa than in Baltic ringed seals, which in turn is approaching that of the leopard seal. *P*-values between Saimaa sample and the other three are from 0.001 to 0.008 (one-tailed Mann–Whitney *U* test, (*SI Appendix*, Table S7). To illustrate the tongue shapes, colors in (*A*) were adjusted to be similar. [Scale bars for (*A*) and (*C*), 5 mm.] Illustrated tooth row profiles in (*C*) are from UEF821, KS.KN47190, NMV13866, and NRM 895081 for Saimaa, Baltic, *Hydrurga*, and *Lobodon,* respectively.

Because many seals swallow food with little or no processing, their tongue plays an important role in feeding, especially in species with a suction feeding strategy (36). Although the availability of soft tissue anatomy is generally limited, next we examined the overall tongue morphology as it may help to explain the distinctive dental morphology of Saimaa ringed seals (Fig. 5*A* and *SI Appendix*, Table S6). We observed that the shape of the tip of the tongue differs between the Saimaa and Baltic ringed seals, and only the former shows intermolar elevations on the lateral sides of the tongue (asterisks in Fig. 5*A*). The width of the tongue is already over 70% of the final width at 20% from the tongue tip in the Saimaa specimens, whereas the Baltic specimen has much acute shape, reaching 70% of the width closer to 40% from the tongue tip (Fig. 5*B* and *SI Appendix*, Table S6). The tongue anatomy was recently examined in Antarctic seals (40) of which the crabeater seal (or the krill-eater seal, *Lobodon carcinophaga*) and the leopard seal (*Hydrurga leptonyx*) are of special interest in comparison to ringed seals. The crabeater seal is a suction and filter-feeding specialist that uses its complex teeth to retain the krill in the oral cavity. Its overall tongue shape appears to be an intermediate between the Saimaa and Baltic ringed seals (Fig. 5*B*). In contrast, the leopard seal tongue is quite similar in overall shape to that of the Baltic ringed seal (Fig. 5*B*). Although leopard seals eat vertebrate prey using a grip-and-tear feeding mode, they seasonally consume krill using a suction and filter-feeding in much the same fashion as crabeater seals (41–43).

The known diets of ringed seals appear to incipiently align within the extremes of larger bodied crabeater and leopard seal diets. Ringed seals in the Arctic feed on small fish, typically around 10 cm, and rarely over 20 cm in length (44). However, Arctic ringed seals also consume large-sized zooplankton as seals in some regions show a seasonal switch to feeding on amphipods such as *Themisto libellula* and other malacostracan crustaceans [(45, 46); see also ref. 47)]. In addition to seasonal feeding, amphipods are also regionally important dietary source for young seals (44). Although data are more limited for the Okhotsk population, also these seals have been reported to eat planktonic crustaceans in addition to fish (47, 48). Apart from the species composition, the Baltic ringed seals have comparable diets with the Arctic (49). In contrast, Lake Saimaa is depauperate of large planktonic crustaceans and its seals feed almost entirely on small fish, such as vendace (*Coregonus albula*), smelt (*Osmerus eperlanus*), and perch (*Perca fluviatilis*) (50, 51).

Taken together, the addition of zooplankton to fish diet and the postcanines with well-developed cuspal comb (34) in marine ringed seals indicate adaptations to the filter feeding of zooplankton somewhat reminiscent of *Lobodon* and *Hydrurga* (Fig. 5*C* and *SI Appendix*, Table S7). In comparison, cusp relief needed for filtering small prey is reduced in Saimaa ringed seals (Fig. 5*C* and *SI Appendix*, Table S7). Rather, Saimaa ringed seals have sharper crown profiles emphasizing the central cusp at the expense of the lateral cusps, especially in the anterior postcanines (Fig. 4). These morphological features suggest increased specialization to catching, biting, and piercing of fish prey. This dental change is unlikely to be the result of a recent drift due to small population size because the broadened tongue implies integrated changes in the feeding apparatus. Kinematic analysis of feeding of marine ringed seals has shown them to be able to suction feed despite the lack of apparent specializations to suction in the skull morphology (52). Considering the oral anatomy, we postulate that Saimaa ringed seals should have relatively strong suction feeding performance. Additional changes linked to feeding are suggested by subtle differences between jaw musculature between Saimaa and Baltic ringed seals (53), and the relative length of the intestinal tract of the Saimaa ringed seal being approximately 20% shorter than those of the Arctic ringed seals (50). Interestingly, the Ladoga ringed seals have not been documented to feed on zooplankton [(54), but see ref. 47], yet their teeth retain the typical ringed seal morphology (Fig. 4). This suggests that the derived oral anatomy of the Saimaa ringed seal reflects a long regime of natural selection. To this end, the deep evolutionary roots of the Saimaa lineage have led to an opposite outcome compared to another long-isolated landlocked seal, the Baikal seal (*Pusa sibirica*). These seals have highly complex teeth, well suited for complementing their fish diet with suction-filter feeding of Lake Baikal’s abundant zooplankton (34, 38, 55).

### Revisiting the Species Status: Saimaa Seal, *P. saimensis* New Rank.

Molecular and morphological data show that Saimaa ringed seals form a distinct, long-divergent lineage, fitting the Genealogical Concordance Species Concept (56). Following similar reasoning used for a diverse range of mammalian taxa (57–60), we propose elevating the Saimaa ringed seal to full species status.

### Systematics.

**Pusa* saimensis* (Nordqvist, 1899) stat. nov.
*Phoca foetida v. saimensis* Nordqvist, 1899
*Phoca hispida ssp. saimensis* Smirnov, 1929

The two closely related genera *Phoca* Linnaeus, 1758 and *Pusa* Scopoli, 1777 distinction has been unclear, with *Pusa* briefly considered a subgenus of *Phoca* (61). Confusion arose as the type species of *Phoca* is the harbor seal (*Phoca vitulina*), while *Pusa*’s original type species, *Phoca foetida* Fabricius, 1776, was later recognized as a synonym of *Phoca hispida* (Schreber, 1775) (62). Currently, *Pusa* is valid for three species: ringed seal (*P. hispida*), Caspian seal (*P. caspica*), and Baikal seal (*P. sibirica*), while *Phoca* includes the harbor seal and spotted seal (*P. largha*) (63). Five subspecies of the ringed seal are recognized, including *P. h. hispida, P. h. botnica, P. h. ochotensis, P. h. ladogensis,* and *P. h. saimensis* (63). While originally described as a form (varia) of the ringed seal (7), the first use of the name as a subspecies combination is attributable to Smirnov (64).

### Etymology.

Nordqvist named the seal after its type locality, Lake Saimaa in Finland (7).

### Lectotype and Paralectotypes.

**Fig. 6.**
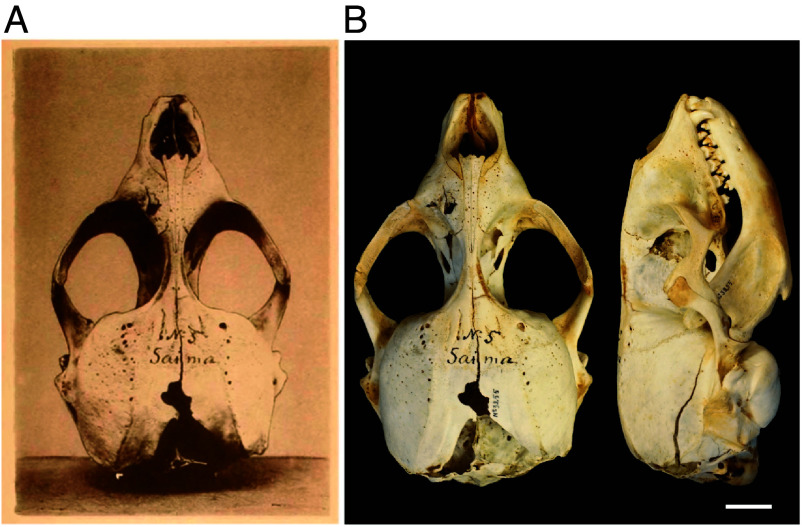
The Saimaa ringed seal (*P. saimensis*) lectotype. (*A*) The skull illustrated by Nordqvist (Tafl. I, in ref. 7). (*B*) The same skull in MZH collections (http://id.luomus.fi/KS.KN3255). Note the blunt force trauma in the skull. [Scale bar for (*B*), 20 mm.]

The skull (KN3255; http://id.luomus.fi/KS.KN3255) illustrated in the original publication (7) is deposited in the zoological collections of the Finnish Museum of Natural History, Helsinki (MZH) (Fig. 6). In addition to this specimen, six other Saimaa ringed seal skulls in the collection have been marked as syntypes (KN2296-KN2299, KN50999). Based on the scant information in (7), their locality has been estimated to be South Savo, Rantasalmi, Saimaa, N61.91384-62.243549, E27.861304-28.739313. Despite damage to the posterior parietal lobes, superior occipital lobe, and behind the maxillae, the skull is in excellent condition, with only the left upper M^1^ missing. All teeth are present on the intact mandible, and the M_1_/P_4_ ratio is 0.83 on both sides. Given its importance in the original description and clear documentation, we designate this syntype specimen as the lectotype for the species. Following the Art. 74.1.3 of the code (65), the other specimens of the syntype series thus become paralectotypes.

Of the six paralectotypes, KN2296 (http://id.luomus.fi/KS.KN2296) has maxillae with all but the right canine and three incisors while the mandibles contain a canine and one premolar (P_4_) on the left side and canine, one premolar (P_4_) and one molar (M_1_) on the right. KN2297 (http://id.luomus.fi/KS.KN2297) lacks mandibles and only the upper left canine and two left posterior incisors (I^2^, I^3^) are present. KN2298 (http://id.luomus.fi/KS.KN2298) has separated mandibles and, apart for the I^2^ on the right maxilla, has all teeth present with the M_1_/P_4_ ratio of 0.84 and 0.86 for left and right side, respectively. KN2299 (http://id.luomus.fi/KS.KN2299) has separated mandibles with teeth present and the M_1_/P_4_ ratio of 0.75 and 0.80 for left and right side, respectively. KN50999 (http://id.luomus.fi/HT.34227) has broken into 10 parts with two loose teeth and remnants of soft tissue.

### Referred Specimens.

We are referring the genomic (*SI Appendix*, Table S2) and morphological specimens (*SI Appendix*, Tables S3 and S6) to Nordqvist’s “saimensis” based on the morphology of their skulls, their anatomy, and their presence in the closed endemic type locality of Lake Saimaa.

### Differential Diagnosis.

The skull of *P. saimensis* has larger mandible, zygomatic width, and jugal length than the other *P. hispida* subspecies (12). Additionally, *P. saimensis* is different from *P. h. botnica* and *P. h. ladogensis* by having higher tympanic bullae and slightly larger orbits (7, 9, 12). The tooth row of *P. saimensis* is shorter than in the other *P. hispida* subspecies (12). The dentition is simpler (7), the lower postcanines lacking five-cusped teeth that are present in low percentages in the other *P. hispida* subspecies (Fig. 4*A*). The M_1_/P_4_ length ratio of 0.88 or below (Fig. 4*E*) is diagnostic for *P. saimensis*, especially for females (*SI Appendix*, Fig. S3). The M_1_ is anteroposteriorly short, even shorter than in the otherwise small *P. h. ochotensis* (Fig. 4*B*).

*P. saimensis* is distinct from all four remaining *P. hispida* subspecies (Arctic, Ladoga, Baltic, and Okhotsk) based on previous and new mtDNA trees [(15, 16), *SI Appendix*, Fig. S1]. In nuclear genome data, *P. saimensis* contains the highest fraction of private variants (n = 37,365; Fig. 2*C*). In a comparison of larger numbers of ringed seals from the Arctic, Baltic, and Ladoga (20), *P. saimensis* is distinguished by 6,495 fixed unique alleles (out of 9,828,400 SNPs).

### Description.

*P. saimensis* is a relatively small seal, the mean body length and mass are 132 cm and 59 kg for females, with males being slightly larger (66). Newborn pups are 68 cm long and weigh 5 kg on average (66), with gray, curly lanugo hair that molts into short, bristly adult-like fur by weaning. *P. saimensis* skulls are characterized by being relatively wide and short (7, 9, 12, 16). Overall, morphometric analyses of cranial morphology exhibit consistent differences between *P. saimensis* and *P. hispida* subspecies (9, 12, 16). The dentition of *P. saimensis* shows emphasis of pointier and taller anterior postcanines at the expense of well-developed cuspal comb found in *P. hispida* subspecies (Figs. 4 and 5). The tongue is relatively broad with a rounded bifurcated tip (Fig. 5*A*).

### Distribution.

The Saimaa seal inhabits Lake Saimaa (61°05′ to 62°36′N, 27°15′ to 30°00′E) covering 4,400 km^2^ with a complex network of basins and narrow straits. The lake is about 180 km long, 140 km wide, with a mean depth of 12 m and a maximum depth of 85 m, and contains roughly 14,000 islands, adding to its labyrinthine structure. The seal population has historically been strongest in the central Haukivesi and Pihlajavesi basins, but recent growth has led to sightings throughout most of the lake.

### Nomenclatural acts.

This published work and the nomenclatural acts it contains are registered in ZooBank (http://zoobank.org), the online registration system for the International Commission on Zoological Nomenclature. The ZooBank Life Science Identifier for this publication is urn:lsid:zoobank.org:pub:A727F0AD-0127-4A4B-9631-B0E1F4AC6ECB.

## Discussion

Genome-wide analyses of species relationships are providing more detailed, and increasingly more complex views into the history of many mammalian lineages (e.g., refs. 57–60). This can complicate taxonomic certainty in delineating species, and approaches integrating multiple lines of evidence have been advocated (67, 68). For example, the traffic-light system proposed by Kitchener et al. (68) uses three types of independent evidence: morphological, genetic, and biogeographical. Accordingly, a simple genetic distance alone, even if large, does not provide taxonomic certainty. Integrative analyses combining multiple lines of evidence have shown that they can result in a reduction of the number of recognized species (e.g., ref. 69) but also in identification of new taxa (e.g. refs. 70 and 71). Given that the Lake Saimaa has formed a geographic barrier for dispersal during the last 10 kya, here we focused on genetic and morphological evidence to examine whether the ancestry of the Saimaa seals could be older than Lake Saimaa.

Our genetic analyses confirm that the seals in Lake Saimaa are not only clearly different from other ringed seals, but importantly, also have an independent and deeper evolutionary history. The Saimaa lineage has twice as distant split times from the Arctic, Baltic, and Ladoga ringed seals than those have among them. Similarly, the Saimaa population has the greatest fraction of unique SNPs, and the PCA component separating the Saimaa individuals from all the ringed seals explains nearly three times the amount of variance that the second component separating the four remaining ringed seal subspecies. Although our analyses reveal a brief period of more recent contact between the Saimaa lineage and the lineages forming the current Ladoga and Baltic populations around 8 to 10 kya, the existence of this gene flow is not contradictory for species separation. In fact, one result of the rapid development of genomic approaches is the recurrent observation of genetic admixture and introgression among many bona fide species in the wild (57, 58, 60).

The deep genetic origin of the Saimaa lineage is further underscored by the morphological data. The distinct skull morphology of Saimaa ringed seals has been long recognized (e.g. ref. 12), and here we demonstrate that their dental and tongue morphologies are indicative of specialization on feeding exclusively on fish. This specialized feeding niche differs from the other ringed seals that have more complex dental morphologies reflecting the ability to include zooplankton in their diet. Taken together, given the combination of genomic and phenotypic evidence, we propose revising the taxonomic rank of the Saimaa ringed seal from a subspecies to a full species status.

The genetic differences and the timing of the population splits (Fig. 3*C* and Table 1) are consistent with the scenario where the ancestral Saimaa population was formed from ice-dammed marine ringed seals in an eastern or southeastern refugium or possibly in multiple refugia latest during the Middle Weichselian, 90 to 50 kya ago, when large glacial lakes existed in the West Siberian Plains and the White Sea Basin with water routes to the eastern edge of the Fennoscandian ice sheet (26–28). We note that although ancient, as a landlocked marine species derived from eastern proglacial refugia, Saimaa seal is not unique. Relict marine fish and invertebrate species are found in modern Lake Saimaa and eastern lakes around the Baltic Sea basin (72–77). It is conceivable that many of the other relicts may also have more ancient origins than previously thought, harboring unique evolutionary history (e.g. ref. 78).

Although Nordqvist (7) originally described the Saimaa and Ladoga ringed seals, he also advocated for the extermination of both populations, due to their claimed nuisance to local fisheries (79). His goal of eradicating the Saimaa seal was nearly realized, with fewer than 100 individuals remaining when the species finally gained legal protection in 1955 (6). The population has gradually recovered since then, reaching almost 500 individuals in 2024 (5). As Saimaa seals depend on snow lairs for birthing and nursing, ongoing anthropogenic climate change presents a significant long-term threat (80). However, the Saimaa seal has survived the previous postglacial warm period and is likely able to do so in the future, if given the chance.

## Materials and Methods

### Material Examined.

DNA samples from ringed seals were obtained from 12 unique sampling localities in the northern hemisphere, greatly improving the coverage in the eastern regions compared to the previous analyses [Fig. 1, distribution ranges drawn after (2), *SI Appendix*, Tables S1 and S2]. The sample sources are listed in *SI Appendix*, Table S2, of which the majority being described in refs. 20 and 78. The sources of the new samples for Baltic and Ladoga are the same as in (20). Alaska samples are from Prudhoe Bay (001B98, AF7110), Peard Bay (G003), and Bering Sea TV13-15); Chukchi Sea samples are from Lorino; Sea of Okhotsk samples are mainly from Chkalov Island and Odyan Bay (TV16-18); Pechora Sea samples are from Vaigach Island; and White Sea samples from Belomorsk archipelago. Samples are from bycaught seals, legally hunted, or dead stranded animals collected for research. Based on earlier analyses (20, 78), the whole-genome-sequenced individuals were ranked by quality (maximizing the sequencing coverage and data completeness) and at most five individuals from each population were included for full genome analyses (in total 46 samples; *SI Appendix*, Table S1). Four spotted seals (*Phoca largha*; accession numbers SAMN08238620, SAMN16895771, SAMN31577600, and SAMN31577601 (81–83) were included for SNP polarization (*SI Appendix*, Table S2). The mtDNA was analyzed separately and 162 unique ringed seal haplotypes and the four spotted seal genomes were included (*SI Appendix*, Table S2). For the dental data (*SI Appendix*, Table S3), Saimaa material are from the University of Eastern Finland (UEF) and Metsähallitus (the Finnish Forest Administration), Ladoga material from Finnish Museum of Natural History (MZH), University of Helsinki, UEF and Metsähallitus, Baltic material from MZH, Arctic Greenland material (East and West Greenland, including Canadian Arctic Archipelago) from National History Museum of Denmark (NHMD), Arctic Alaska material (Beaufort, Chukchi, and Bering Sea) from University of Alaska Museum of the North, and Arctic Kara Sea and Sea of Okhotsk material from the National Museum of Nature and Science of Tokyo. The four distal postcanines show the largest range of variation among phocid species, and their intra- and interspecies variation have been studied extensively previously (34, 84, 85). Additionally, we investigated soft tissue anatomy of tongues from freshly defrozen seal cadavers (n = 8, *SI Appendix*, Table S6, from Metsähallitus (the Finnish Forest Administration). The type specimens are presented in a separate chapter. Comparative dental specimens to calculate cusp relief (*SI Appendix*, Table S7) originated from the collections of the MZH, the Swedish Museum of Natural History (NRM), and the National Museum of Victoria, Melbourne (NMV).

### Data Mapping and Nuclear Genome Variant Calling.

The DNA sequencing was performed on Illumina NextSeq500 and NovaSeq 6000 platforms (20, 21). Read data for ringed seal samples and four spotted seal samples (*SI Appendix*, Table S2) were aligned as described previously (86). For the selected 46 ringed seals and four spotted seals, variants were called by applying GATK4 (v.4.2.5) (87) HaplotypeCaller for each sample, joining the data with CombineGVCFs, and applying GenotypeGVCFs to the joint data. The sequencing depth of the studied ringed seal samples varied from 5.0× to 29.5× with the mean coverage of 10.6×, and of spotted seal samples from 34.8× to 115.7× with the mean coverage of 55.7×.

### Phylogenetic Analyses.

Variants within the mitochondrial genome were called from all sequenced individuals applying the same GATK4 tools but defining the ploidy 1. Protein-coding regions were inferred by mapping the peptide sequences of the *Pusa hispida* mitochondrion (NC_008433) against the reference mtDNA contig with miniprot (88). Binary SNP variants, totaling 1,434, within the CDS regions were extracted and samples with missing data were discarded. The VCF data were converted to FASTA sequences using the vcf-to-tab tool from the VCFtools package (89) and the script from https://github.com/JinfengChen/vcf-tab-to-fasta modified to work on haploid data. Identical haplotypes (all within a subspecies) were removed, leaving 162 ringed seals and four spotted seals as the outgroup (*SI Appendix*, Table S2). The phylogenetic tree was inferred with RAxML v.8.2.12 (90) using the model ASC_GTRGAMMA and correcting for the invariable sites (totaling 9,945) missing from the VCF file with the option --asc-corr=felsenstein. For the search, the rapid option combining the ML search (20 replicates) and the bootstrap analysis (1,000 replicates) was selected (-f a -# 1000). The resulting tree was visualized in R using the ggtree package (91).

For phylogenetic analyses of nuclear data, two individuals with the highest sequencing coverage were selected from nine ringed seal populations and from spotted seals, and binary SNP data over positively masked regions (86) were partitioned using a custom script. Sites within 1 Mbp windows were extracted with BCFtools (v.1.9) (89), excluding positions with missing data and leaving 0.5 Mbp gaps between adjacent windows. The VCF data were converted to tabular format with vcf-to-tab tool and to FASTA format with the vcf-tab-to-fasta script, modified to randomly draw one of the heterozygous alleles. The number of invariant sites within each genomic window was calculated from the positive mask and corrected for sites turned invariant in allele sampling. A phylogenetic tree for each window was inferred with RAxML using the model ASC_GTRGAMMA and the option --asc-corr=felsenstein as above. The resulting 1,537 trees were imported to R using the ape package (v. 5.8) (92) and 500 trees were randomly sampled. The trees were rooted with spotted seals which were then removed. The data were visualized with the densiTree function from the phangorn package (v. 2.11.1) (93).

### Data Filtering and SNP Analyses.

The binary SNP data for the full nuclear genomes were filtered and thinned using BCFtools (v.1.9) (89) and Plink (v.1.9) (94). Sites falling outside the positively masked regions or inside the repeat-masked regions (86), showing no variation among ringed seals or having >10% of missing data were excluded. The remaining data, totaling 25,697,406 sites, were further thinned by requiring sites to be at least 1 kbp apart, leaving 1,807,907 variant sites.

A PCA was performed on the thinned data (1.8M sites) with smartpca (v.16000)(95) and the results were visualized with R (96) using the ggplot2 (97) package. In smartpca, the normalization option (usenorm) was turned off and the analysis was performed for the full 46 individuals set and for an even sampling of five individuals per subspecies (22), selected based on data completeness and, in the case of the Arctic subspecies, picking individuals from different geographic locations (Alaska, Svalbard, White Sea, Pechora Sea, Chukchi Sea). Starting from the 1.8M sites data, allele-sharing among different subspecies was studied using the same five individuals per subspecies, including the four spotted seals for derived allele inference. With samples varying from one to five individuals, the presence of a derived allele in each ringed seal subspecies was recorded, and the proportion of derived allele patterns unique to the specific subspecies was calculated. The statistic is highly dependent on the MAF limit applied. With full data (MAF = 0), a large proportion of unique alleles are singletons and reflect the recent demographic events and the current effective population size. We applied MAF = 0.05 (requiring at least five derived alleles among the 92 sampled genomes in the full data) to understand the more distant demographic events; however, one cannot rule out the founder effect and recent genetic drift inflating the Saimaa statistics. The proportion of private variants is expected to decrease with the number of individuals sampled and, in theory, plateau and stop when all individuals are included. The allele frequencies in each subset were computed with VCFtools and processed with bash and R scripts.

### Demographic Analyses.

Demographic analyses were performed with MSMC-IM (25) using the whole-genome data from (20). For within-population analyses, ten two-individual sets (i.e., four chromosomes) were randomly chosen from each population and their coalescent rate histories were computed as explained in ref. 20. For cross-population coalescence rate analyses, six pairs of individuals for each population combination (i.e., two chromosomes from each population, two populations per analysis) were randomly chosen and their coalescent rate histories were computed using the default time segmenting. The combined rate estimates were analyzed using MSMC_IM.py with the recommended parameters and the mutation rate (μ) 1.826e-8 estimated for polar bear (98). We consider this a robust estimate as it falls within the mutation rates estimated for different seals (99) and using different estimates does not affect the overall pattern of results. The summary results were computed and visualized with R using the ggplot2 package and assuming a generation time (*g*) of 10 y.

### Phenotypic Analyses.

Lower tooth rows were photographed from the lingual side and maximum tooth lengths, maximum heights, top-cusp angles, and cusps numbers were tabulated using Fiji (100) and additional analyses were done with PAST (101). Although the dentitions of phocids show high degree of intraspecies variation, the seals are born with their permanent tooth crowns fully formed and in the process of erupting into the oral cavity (102, 103). Therefore, in contrast to other skeletal features, dental measures do not change after birth other than through wear (e.g., 34, 37). We report the analyses for the four, right side postcanines (P_2_, P_3_, P_4_, M_1_), and only for individual specimens from which the measures could be obtained for all the four teeth. We excluded specimens with dental anomalies such as supernumerous teeth. Cusp number and tooth length were measured for 326 individuals and crown height and top-cusp angle for 281 individuals due to cracked or slightly worn specimens (*SI Appendix*, Table S3). Height and angle measurements were estimated for cases where only the enamel cap of the cusp tip was missing. Exclusion of these data did not alter the results. Cusps were tabulated regardless how small they are, but rarely present small cusps on the lingual cingulid were excluded. Relative cusp height was measured using the top-cusp angle (84, 104) and the cusp relief as the length of the crown perimeter divided by the length of the tooth at the crown base. We list also the cusp index measure of crown complexity (34) that standardizes the crown relief by crown height (*SI Appendix*, Table S7). *P*-values in Fig. 3 and *SI Appendix*, Table S5 are one-tailed and obtained between group means using 10,000 permutations. The ROC curves were used to obtain correct classification probabilities. ROC curves have been more typically used for medical data analyses, but can also be applied for taxonomical questions. For tooth lengths and top-cusp angles, smaller values denote true positive. Because the tongue plays an important role in seal feeding, especially in species with a suction feeding strategy (37, 40), we compared the overall morphology of Saimaa ringed seal tongue with that of phocids from the literature (40). Tongues from cadavers were photographed in the defrosted stage and compared to those taken from published photographs of *Lobodon* and *Hydrurga* tongues (40). To obtain a robust measure of shape apart from size and variation caused by differential preservation of soft tissue anatomy, were divided tongue images into ten-percent bins from the tip to the end of the posterior body, and tabulated widths as relative to the maximum width of the tongue.

## Supplementary Material

Appendix 01 (PDF)

## Data Availability

The genome data are available at the European Nucleotide Archive (ENA) under Accession No. PRJEB56329 (105) and PRJEB56317(106). The newly generated 16 whole-genome sequencing datasets are deposited under PRJEB56317 (106) with Accession No. ERS23016468-ERS23016483 (107, 108). Instructions for replicating the computational analyses are provided at https://github.com/ariloytynoja/pusa_saimensis (109). All other data are included in the manuscript and/or *SI Appendix*.
