# Supplementary material for: Deep origins, distinct adaptations, and species-level status indicated for a glacial relict seal
Source: Proc Natl Acad Sci U S A. 2025 Jun 10;122(25):e2503368122. doi: 10.1073/pnas.2503368122 (PMC12207470; doi:10.1073/pnas.2503368122)
Supplement: Supplementary file 1 — Appendix 01 (PDF) [file pnas.2503368122.sapp.pdf]

PNAS

Supporting Information for

Deep origins, distinct adaptations and species level status indicated for a glacial relict seal

Ari Löytynoja[a,1], Jaakko Pohjoismäki[b,1], Mia Valtonen[c], Juha Laakkonen[d], Wataru Morita[e], Mervi Kunnasranta[b,f], Risto Väinölä[g], Morten Tange Olsen[h], Petri Auvinen[i], Jukka Jernvall[i,j,1]

[1]To whom correspondence may be addressed. Email: ari.loytynoja@helsinki.fi, jaakko.pohjoismaki@uef.fi or jernvall@fastmail.fm

This PDF file includes:

Figures S1 to S3
Tables S1 to S7
SI References

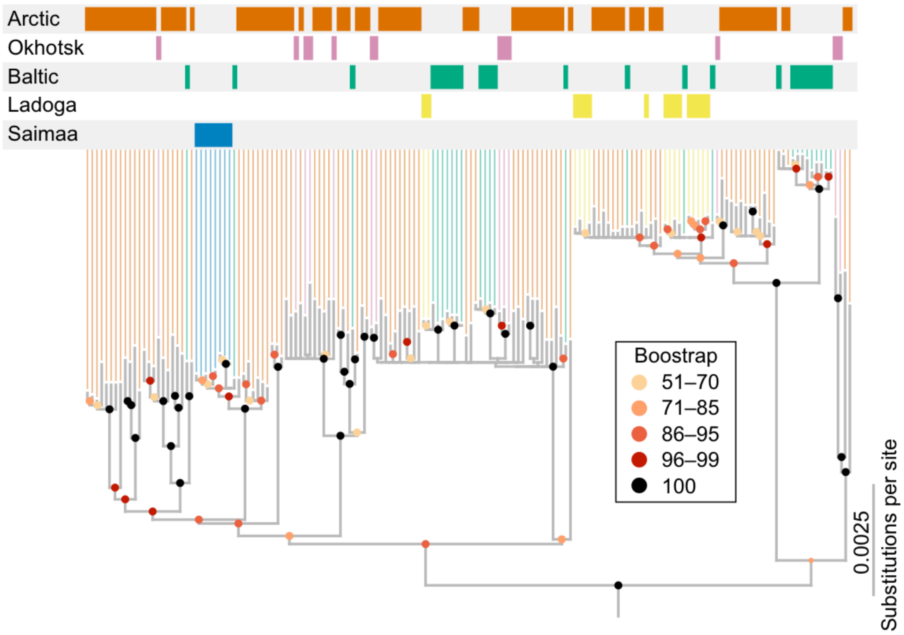

Fig. S1. The maximum likelihood tree for the unique mitogenome haplotypes rooted with four spotted seals. The dots at internal nodes indicate bootstrap support values greater than 50%. In contrast to the four ringed seal subspecies, all Saimaa samples (in blue) cluster tightly together and form an evolutionary lineage of their own.

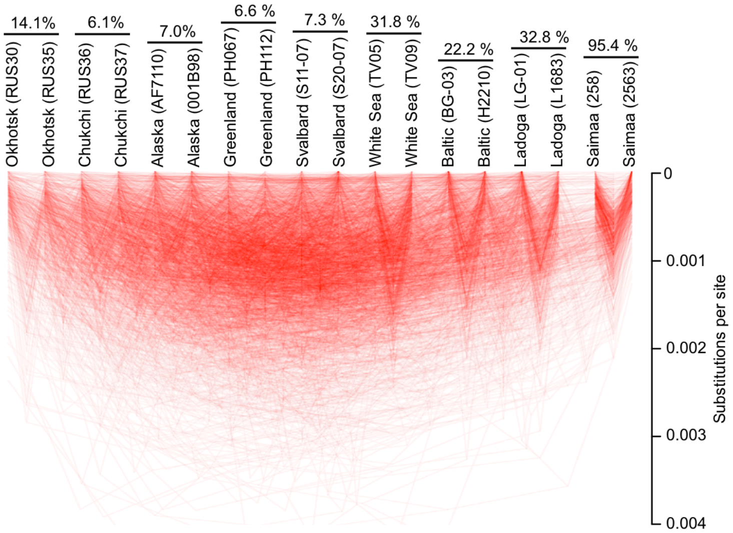

Fig. S2. The distribution of maximum likelihood trees for a random sample of 500 distinct nuclear genome regions, each 1 Mbp wide. Due to recombination and potential admixture of different evolutionary lineages, trees across the genome differ. Although the inferred trees vary greatly, the true variation is expected to be even greater and no single phylogeny reflects the true history for all sites in a window. Nevertheless, Saimaa individuals show relatively few recent links to the other individuals and, among the 1537 genome regions studied, have the highest support for the monophyly (percentage of the genome regions supporting monophyly on the top).

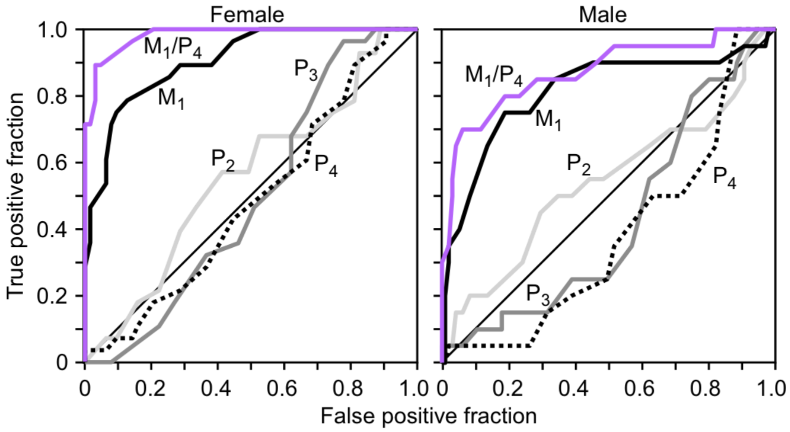

Fig. S3. The receiver operating characteristic (ROC) curves for tooth lengths plotted separately for female and male specimens. Of the 326 specimens measured, 96 (28 from Saimaa) and 115 (20 from Saimaa) are assigned to female and male, respectively. For both sexes, the M_1/P_4 performs the best, but female seals show almost perfect classification. Areas below the M_1 and M_1/P_4 curves are 0.90 and 0.98 for females, and 0.81 and 0.87 for males.

Table S1. Number of ringed seal samples included from each locality. For mtDNA analyses, only unique haplotypes were included. Full listing of the data is in *SI Appendix*, Table S2.

Locality	Full genomes (n)	Mitogenomes (n)
Alaska	5	6
Canada	4	12
Western Greenland	4	37
Eastern Greenland	2	20
Svalbard	5	11
White Sea	2	6
Pechora Sea	1	2
Chukchi Sea	3	3
Okhotsk Sea	5	13
Baltic Sea	5	28
Ladoga	5	16
Saimaa	5	7
Total	46	161

Table S2. Samples included in mtDNA (m), whole-genome (g) or in both (mg) analyses.

Species	Sample ID	Analysis	Origin	Study
Pusa hispida	001B98	mg	Alaska, USA	this study
	AF7110	mg	Alaska, USA	this study
	G003	mg	Alaska, USA	this study
	TV13	mg	Alaska, USA	this study
	TV14	mg	Alaska, USA	this study
	TV15	m	Alaska, USA	this study
	BG-01	m	Baltic Sea, Finland	(1)
	BG-02	m	Baltic Sea, Finland	(1)
	BG-03	mg	Baltic Sea, Finland	(1)
	BG-06	m	Baltic Sea, Finland	this study
	BG-09	mg	Baltic Sea, Finland	(1)
	BG-10	m	Baltic Sea, Finland	(1)
	BG-12	mg	Baltic Sea, Finland	(1)
	H2210	mg	Baltic Sea, Finland	(1)
	N160	m	Baltic Sea, Finland	(1)
	N2-04	m	Baltic Sea, Finland	this study
	N210	m	Baltic Sea, Finland	(1)
	N3-09	m	Baltic Sea, Finland	(1)
	N4-04	m	Baltic Sea, Finland	this study
	N7-07	m	Baltic Sea, Finland	(1)
	N8-07	m	Baltic Sea, Finland	this study
	NN10-07	m	Baltic Sea, Finland	this study
	NN1-06	m	Baltic Sea, Finland	this study
	NN1-07	m	Baltic Sea, Finland	this study
	NN11-07	m	Baltic Sea, Finland	this study
	NN12-07	m	Baltic Sea, Finland	this study
	NN13-07	m	Baltic Sea, Finland	this study
	NN2-07	m	Baltic Sea, Finland	this study
	NN3-07	g	Baltic Sea, Finland	(1)
	NN5-07	m	Baltic Sea, Finland	this study
	NN8-07	m	Baltic Sea, Finland	this study
	NN9-07	m	Baltic Sea, Finland	this study
	PA4912	m	Baltic Sea, Finland	this study
	PA4913	m	Baltic Sea, Finland	this study
	PA8364	m	Baltic Sea, Finland	this study
	PH157	mg	Holman, Canada	(2)
	PH158	m	Holman, Canada	(2)
	PH161	mg	Holman, Canada	(2)
	PH163	m	Holman, Canada	(2)
	PH165	m	Holman, Canada	(2)
	PH169	m	Arviat, Canada	(2)
	PH176	mg	Arviat, Canada	(2)
	PH185	mg	Arviat, Canada	(2)
	PH186	m	Arviat, Canada	(2)
	PH187	m	Arviat, Canada	(2)
	PH190	m	Holman, Canada	(2)
	PH194	m	Holman, Canada	(2)
	RUS36	mg	Chukchi Sea	this study
	RUS37	mg	Chukchi Sea	this study
	RUS38	mg	Chukchi Sea	this study
	PH001	m	Ittoqqortoormiit, Greenland	(2)
	PH002	m	Ittoqqortoormiit, Greenland	(2)
	PH003	m	Ittoqqortoormiit, Greenland	(2)
	PH006	m	Ittoqqortoormiit, Greenland	(2)
	PH013	m	Qaanaaq, Greenland	(2)
	PH015	m	Qaanaaq, Greenland	(2)
	PH016	m	Qaanaaq, Greenland	(2)
	PH017	m	Ittoqqortoormiit, Greenland	(2)
	PH025	m	Qaanaaq, Greenland	(2)

Species	Sample ID	Analysis	Origin	Study
Pusa hispida	PH027	m	Qaanaaq, Greenland	(2)
	PH030	m	Qaanaaq, Greenland	(2)
	PH032	m	Qaanaaq, Greenland	(2)
	PH033	m	Qaanaaq, Greenland	(2)
	PH035	m	Qeqertarsuaq, Greenland	(2)
	PH042	m	Ittoqqortoormiit, Greenland	(2)
	PH043	m	Ittoqqortoormiit, Greenland	(2)
	PH044	mg	Ittoqqortoormiit, Greenland	(2)
	PH046	m	Qaanaaq, Greenland	(2)
	PH047	m	Ittoqqortoormiit, Greenland	(2)
	PH062	m	Qaanaaq, Greenland	(2)
	PH063	m	Qaanaaq, Greenland	(2)
	PH065	m	Qaanaaq, Greenland	(2)
	PH067	mg	Qaanaaq, Greenland	(2)
	PH070	m	Qaanaaq, Greenland	(2)
	PH077	m	Ittoqqortoormiit, Greenland	(2)
	PH081	m	Ittoqqortoormiit, Greenland	(2)
	PH082	m	Ittoqqortoormiit, Greenland	(2)
	PH083	m	Ittoqqortoormiit, Greenland	(2)
	PH085	m	Ittoqqortoormiit, Greenland	(2)
	PH086	mg	Ittoqqortoormiit, Greenland	(2)
	PH087	m	Ittoqqortoormiit, Greenland	(2)
	PH089	m	Ittoqqortoormiit, Greenland	(2)
	PH090	m	Ittoqqortoormiit, Greenland	(2)
	PH091	m	Ittoqqortoormiit, Greenland	(2)
	PH093	m	Qaanaaq, Greenland	(2)
	PH095	m	Ittoqqortoormiit, Greenland	(2)
	PH102	m	Qaanaaq, Greenland	(2)
	PH109	m	Qeqertarsuaq, Greenland	(2)
	PH111	m	Qeqertarsuaq, Greenland	(2)
	PH112	mg	Qeqertarsuaq, Greenland	(2)
	PH113	m	Qeqertarsuaq, Greenland	(2)
	PH114	m	Qeqertarsuaq, Greenland	(2)
	PH115	m	Qeqertarsuaq, Greenland	(2)
	PH116	mg	Qeqertarsuaq, Greenland	(2)
	PH121	m	Kangia, Greenland	(2)
	PH122	m	Kangia, Greenland	(2)
	PH123	m	Kangia, Greenland	(2)
	PH124	m	Kangia, Greenland	(2)
	PH125	m	Kangia, Greenland	(2)
	PH126	m	Kangia, Greenland	(2)
	PH127	m	Kangia, Greenland	(2)
	PH130	mg	Kangia, Greenland	(2)
	PH135	m	Kangia, Greenland	(2)
	PH136	m	Kangia, Greenland	(2)
	PH137	m	Kangia, Greenland	(2)
	PH146	m	Kangia, Greenland	(2)
	PH147	m	Kangia, Greenland	(2)
	L1501	mg	Ladoga, Russia	(1)
	L1683	mg	Ladoga, Russia	(1)
	L1-92	m	Ladoga, Russia	this study
	L1-94	m	Ladoga, Russia	this study
	L2195	m	Ladoga, Russia	this study
	L2202	m	Ladoga, Russia	this study
	L2308	m	Ladoga, Russia	this study
	L2-94	mg	Ladoga, Russia	this study
	L3-94	m	Ladoga, Russia	this study
	LG-01	mg	Ladoga, Russia	(1)
	LG-02	mg	Ladoga, Russia	(1)
	LG-04	m	Ladoga, Russia	(1)

Species	Sample ID	Analysis	Origin	Study
Pusa hispida	LG-06	m	Ladoga, Russia	(1)
	LM12	m	Ladoga, Russia	this study
	LM16	m	Ladoga, Russia	this study
	LM17	m	Ladoga, Russia	this study
	RUS18	m	Okhotsk Sea, Russia	this study
	RUS19	m	Okhotsk Sea, Russia	this study
	RUS28	m	Okhotsk Sea, Russia	this study
	RUS29	mg	Okhotsk Sea, Russia	this study
	RUS30	mg	Okhotsk Sea, Russia	this study
	RUS31	m	Okhotsk Sea, Russia	this study
	RUS32	m	Okhotsk Sea, Russia	this study
	RUS33	m	Okhotsk Sea, Russia	this study
	RUS34	m	Okhotsk Sea, Russia	this study
	RUS35	mg	Okhotsk Sea, Russia	this study
	TV16	mg	Okhotsk Sea, Russia	this study
	TV17	m	Okhotsk Sea, Russia	this study
	TV18	mg	Okhotsk Sea, Russia	this study
	TV10	m	Pechora Sea, Russia	this study
	TV11	mg	Pechora Sea, Russia	this study
	258	g	Saimaa, Finland	(1)
	292	g	Saimaa, Finland	(1)
	1090	m	Saimaa, Finland	(1)
	1235	m	Saimaa, Finland	(1)
	1482	m	Saimaa, Finland	(1)
	1956	m	Saimaa, Finland	(1)
	1957	m	Saimaa, Finland	(1)
	1977	m	Saimaa, Finland	(1)
	1983	g	Saimaa, Finland	(1)
	2333	m	Saimaa, Finland	(1)
	2504	m	Saimaa, Finland	(1)
	2563	g	Saimaa, Finland	(1)
	2614	g	Saimaa, Finland	(1)
	S10-07	m	Svalbard, Norway	(2)
	S11-07	mg	Svalbard, Norway	(2)
	S11-96	m	Svalbard, Norway	(2)
	S15-96	m	Svalbard, Norway	(2)
	S18-96	m	Svalbard, Norway	(2)
	S19-07	m	Svalbard, Norway	(2)
	S20-07	mg	Svalbard, Norway	(2)
	S4-07	m	Svalbard, Norway	(2)
	S6-07	mg	Svalbard, Norway	(2)
	S7-07	mg	Svalbard, Norway	(2)
	S8-07	mg	Svalbard, Norway	(2)
	TV05	mg	White Sea, Russia	this study
	TV06	m	White Sea, Russia	this study
	TV07	m	White Sea, Russia	this study
	TV08	m	White Sea, Russia	this study
	TV09	mg	White Sea, Russia	this study
	WS1-14	m	White Sea, Russia	this study
Phoca largha	SAMN08238620	mg	Gangwon-do, South Korea	(3)
	SAMN16895771	mg	NIST Biorepository	(4)
	SAMN31577600	mg	Liaodong Bay, China	(5)
	SAMN31577601	mg	Liaodong Bay, China	(5)

Table S3. Specimens and measurements used in the analyses of dental variation.

Population	Sex	Cusp number (n)				Length (mm)				Height (mm)				Angle (degrees)			
ID		P_2	P_3	P_4	M_1	P_2	P_3	P_4	M_1	P_2	P_3	P_4	M_1	P_2	P_3	P_4	M_1
Saimaa, n = 50																	
107	m	4	4	4	4	7.0	7.5	7.1	7.2	5.7	6.1	5.4	4.5	101	100	110	119
220	f	4	4	4	3	6.3	6.7	6.5	5.3	5.0	5.0	4.9	3.9	93	103	112	111
291	m	3	4	4	3	5.4	5.9	6.2	5.2	5.1	5.3	4.7	3.9	100	92	111	116
427	f	4	4	4	3	6.3	6.7	6.6	5.8	5.5	5.6	4.9	4.4	86	103	112	122
759	m	4	4	4	3	6.6	6.9	6.5	5.8	5.5	5.7	5.1	4.5	94	99	115	112
821	f	4	4	4	3	5.9	6.3	6.3	4.5	4.9	4.8	4.3	3.8	87	103	112	106
824	f	3	4	4	3	5.5	6.8	6.8	5.8	4.7	5.4	4.9	4.1	98	104	121	119
890	f	4	4	4	3	5.0	5.8	6.0	5.1	5.0	5.2	4.9	4.2	83	95	99	110
1121	m	4	4	4	4	6.7	6.9	6.8	5.6	5.6	5.9	5.1	4.1	88	99	109	123
1191	f	4	4	4	3	5.5	6.1	6.1	4.3								
1234	m	3	4	4	3	5.2	6.7	6.8	5.7	4.7	5.2	4.8	4.0	101	101	113	114
1269	m	4	4	4	3	6.0	6.6	6.8	5.8	5.0	5.6	5.0	4.4	87	102	115	112
2222	f	4	4	4	3	6.1	6.6	6.6	4.4								
2259	f	4	4	4	3	5.7	6.5	6.2	5.2	5.0	5.3	5.0	3.9	94	95	107	105
2341	f	4	4	4	3	4.9	6.1	6.1	5.4	4.8	4.9	4.8	4.0	86	102	112	114
2342	m	4	4	4	3	6.6	6.4	6.9	6.0	5.3	5.6	5.2	4.2	94	105	119	113
2343	f	4	4	4	4	6.2	6.7	6.7	5.6	5.6	5.3	4.9	4.2	92	102	111	113
2348	m	3	4	4	4	5.7	6.8	6.9	6.6	5.2	4.9	4.9	4.1	90	109	111	114
2379	f	4	4	4	3	5.5	6.4	6.7	4.4	4.9	5.6	5.1	3.7	102	107	116	111
2386	f	3	4	4	3	5.3	6.8	6.8	5.9	5.0	4.5	3.8	4.7	97	98	111	121
2396	m	3	4	4	3	5.6	7.3	7.0	5.6	5.5	5.6	5.0	4.4	89	110	125	111
2397	f	3	4	4	4	5.9	6.1	6.4	5.0	5.2	5.5	4.8	4.1	88	98	116	105
2433	f	4	4	4	3	5.3	6.0	5.0	3.9	4.9	5.1	4.3	3.4	91	101	111	104
2436	m	3	3	4	3	4.5	5.4	5.5	5.2	4.6	5.0	4.6	3.9	83	83	95	109
2440	m	4	4	4	3	5.8	6.6	6.6	5.0	5.4	5.6	5.3	4.0	95	97	113	112
2445	f	4	4	4	3	6.3	6.5	6.5	5.2	5.4	5.5	5.2	4.3	93	101	113	112
2448	f	4	4	4	4	5.9	6.5	6.4	5.2								
2451	f	3	4	4	4	5.6	6.7	6.7	5.5								
2506	m	4	4	4	4	5.6	6.1	6.6	6.1	5.7	5.7	5.2	4.8	83	99	111	111
2509	f	4	4	4	3	6.1	6.3	6.2	5.1	5.4	5.5	5.1	4.1	91	96	103	111
2510	m	4	4	4	3	5.7	6.9	6.5	5.4								
2513	m	4	4	4	3	6.2	6.4	6.6	5.7	5.5	6.0	5.4	4.5	87	94	109	108
2519	m	4	4	4	3	6.8	7.2	7.1	5.5	5.3	5.8	5.3	4.2	91	97	104	104
2534		4	4	4	4	5.8	6.5	5.9	5.2								
2549	f	4	4	4	3	6.3	6.4	6.2	4.5	5.6	5.8	4.9	4.1	91	97	108	95
2555	f	3	4	4	3	5.5	6.1	5.6	4.6	4.9	5.3	4.7	4.0	100	96	105	108
2557	m	4	4	3	3	6.3	6.9	6.2	5.1	5.7	5.9	5.2	4.0	92	103	110	115
2584	f	4	4	4	3	6.5	6.6	6.3	5.0	5.5	5.3	4.8	4.2	99	109	112	116
2585	f	4	4	4	3	5.7	6.2	5.8	4.9	4.8	4.7	4.5	3.7	93	106	116	116
2586	m	3	4	4	3	5.2	6.7	6.3	5.3	5.3	5.6	4.9	4.1	92	100	116	116
2588	m	4	4	4	3	6.9	6.7	7.0	5.3	5.9	5.8	5.4	4.4	94	100	116	115
2591	f	4	4	4	3	5.6	6.4	6.3	4.9	5.5	5.4	5.0	4.0	87	97	111	111
2593	m	4	4	4	3	6.2	7.0	7.0	6.0	5.3	5.3	4.9	4.1	90	112	126	118
2597	f	4	4	4	3	5.7	5.9	6.2	4.9	5.1	5.4	4.8	3.9	89	99	114	114
2606	f	4	4	4	3	6.5	7.0	6.9	5.5	6.0	5.7	5.3	4.4	91	104	117	105
2610	f	4	4	4	3	5.4	6.0	5.9	4.8	5.4	5.3	4.5	3.8	90	100	114	122
2611	m	3	4	4	3	5.7	6.7	6.4	5.7	4.9	5.3	4.9	4.0	94	100	116	116
2614	f	3	3	4	3	5.3	5.9	5.9	4.8	5.2	5.5	5.3	4.1	80	94	102	102
2615	f	4	4	4	4	5.5	6.3	6.5	4.4	5.5	5.7	4.9	4.0	77	89	112	96
2619		3	4	4	3	5.6	6.6	6.5	5.5	5.6	5.8	5.4	4.2	85	91	106	114
Ladoga, n = 67																	
1160		4	4	4	3	6.1	6.6	6.7	5.8	5.2	5.1	5.0	4.1	97	110	116	122
1163		4	4	4	4	5.8	6.3	6.3	6.8	5.4	5.5	5.2	4.8	91	102	115	121
1188		4	4	4	3	6.3	7.0	6.7	6.8	5.3	5.5	5.3	4.5	98	115	114	122
1197		4	4	4	4	6.5	6.3	6.0	5.7	4.7	5.1	4.9	4.0	104	105	109	116
1199		4	4	4	3	7.3	7.6	7.5	7.0	5.1	5.4	5.2	4.4	106	108	119	134
1212		4	4	4	4	6.2	6.5	6.4	6.1	4.8	5.1	4.7	3.6	108	112	115	122
1215		4	4	4	3	6.0	6.8	6.9	6.1	4.7	5.2	4.9	4.2	104	114	121	110
1218		4	5	5	5	6.3	7.1	6.6	6.6	5.4	5.4	5.4	4.1	100	110	109	128
1222		4	4	4	4	6.5	6.7	6.9	5.9	5.5	5.2	5.0	3.7	90	104	110	121
1225		4	4	4	3	6.4	7.1	7.1	5.9	5.8	5.6	5.3	4.0	92	108	114	123
1226		4	4	5	4	6.8	7.5	7.9	6.4	5.2	5.4	5.0	4.0	99	112	118	120
1234		4	4	4	3	5.7	6.2	6.7	6.5	5.2	5.1	4.9	4.4	90	98	109	126
1236		4	4	4	4	5.7	6.2	6.4	6.3	4.8	5.4	4.8	4.4	105	106	119	120
1237		4	4	4	3	6.6	6.8	6.9	5.0	5.5	5.6	5.5	3.8	97	110	113	117

Population	Sex	Cusp number (n)				Length (mm)				Height (mm)				Angle (degrees)			
ID		P_2	P_3	P_4	M_1	P_2	P_3	P_4	M_1	P_2	P_3	P_4	M_1	P_2	P_3	P_4	M_1
1238		4	4	4	4	6.4	7.3	7.4	6.6	5.3	5.7	5.6	4.7	106	113	118	130
1239b		4	4	4	4	6.9	6.9	6.7	6.2	5.9	5.9	5.1	4.6	102	103	109	111
1240		4	4	4	4	5.1	5.2	5.4	5.6	5.1	5.2	4.9	4.2	92	88	96	121
1241		4	4	4	3	6.2	6.8	6.4	5.4	5.1	5.4	5.0	3.6	99	109	111	125
1242		4	4	4	4	7.1	7.3	7.3	7.4	5.5	5.8	5.5	4.8	104	107	109	123
1245		4	4	4	3	6.4	7.1	7.4	6.6	5.2	5.3	4.9	4.3	93	112	118	119
1247		4	4	4	3	5.9	6.4	6.1	5.8	4.9	5.2	4.9	4.4	97	101	104	112
1249		4	4	4	4	6.5	6.8	6.8	6.3	4.7	5.1	5.0	4.1	111	113	112	117
1250		4	4	4	4	5.9	6.3	6.0	5.8	4.8	4.8	4.4	3.5	90	94	114	124
1251		4	5	5	5	6.0	6.7	7.3	6.3	5.0	5.0	4.7	3.9	106	111	116	127
1253		4	4	4	4	6.1	6.5	6.5	6.4	4.9	5.3	5.1	4.7	117	107	108	118
1254		4	4	4	3	6.3	6.8	6.9	6.9	5.0	5.6	5.2	4.6	97	104	105	119
1259		4	4	4	3	6.4	7.1	7.1	6.3	5.0	5.5	5.3	4.3	104	102	113	129
1260		3	4	4	3	5.3	6.2	6.1	5.9	5.1	4.7	4.4	3.6	88	99	112	118
1261		4	4	4	3	5.5	6.0	5.9	5.3	4.7	5.1	4.7	3.7	88	91	101	111
1262		4	4	4	4	6.0	6.5	6.2	6.0	5.0	4.8	4.9	4.2	103	113	112	121
1266		4	4	4	4	5.7	6.1	6.0	5.9								
1268		4	4	4	3	6.3	6.7	6.5	5.6								
1269		4	4	4	4	5.7	6.8	6.5	6.1	4.9	5.1	4.8	4.0	95	119	117	132
1275		4	4	4	3	5.3	6.2	6.1	6.1	4.5	5.1	4.8	4.4	81	91	104	111
1276		4	4	4	4	5.5	6.0	6.1	6.4	4.7	5.1	4.9	4.2	97	103	107	117
1277		4	4	4	4	5.9	6.3	6.0	6.2	4.8	5.2	4.8	3.9	105	107	118	133
1278		4	4	4	3	6.3	6.9	7.0	5.8	5.7	5.6	5.1	4.1	80	96	106	113
1279		4	4	4	4	5.8	6.0	6.1	6.1	5.2	4.9	4.3	4.1	94	113	124	119
1385a		4	4	4	3	6.0	6.8	6.3	5.6								
1501		4	4	4	4	6.0	6.9	7.0	6.5	4.7	5.4	4.9	4.0	97	102	112	121
1927		4	4	4	4	6.0	6.8	7.0	6.5								
2104		4	4	4	4	6.7	7.2	6.7	6.7	5.2	5.3	5.1	4.4	107	116	121	129
2105		4	4	4	3	6.4	6.8	6.7	5.4	5.3	5.2	5.2	4.2	98	106	109	118
2120		4	4	4	4	5.6	6.1	6.1	6.1	4.8	5.0	4.8	4.5	91	106	112	117
2121		4	4	4	5	6.5	6.7	6.9	6.3	5.3	5.5	5.3	4.0	103	109	107	115
2123		4	4	4	3	6.6	6.7	6.5	6.2	5.6	5.8	5.3	4.4	104	109	109	117
2125		4	4	4	4	6.3	6.2	6.3	6.3	5.1	5.5	5.2	4.6	100	101	105	113
2130		4	4	4	3	6.1	6.8	6.8	5.7	4.6	4.7	4.5	3.5	101	114	115	123
2152		4	4	4	3	5.8	6.3	6.3	5.9	4.8	5.0	4.8	3.9	102	109	110	124
2157		4	4	4	4	6.8	6.9	6.8	6.8	5.4	5.8	5.5	4.6	105	109	113	128
2158		4	4	4	3	5.6	6.0	6.4	5.8	4.6	4.9	4.8	4.4	99	107	115	123
2159		4	4	4	3	6.5	6.8	6.9	6.3	5.6	5.8	5.5	4.8	95	105	116	118
2160		4	4	4	3	5.6	6.2	6.2	5.9								
2177		4	4	4	3	6.7	7.3	7.2	6.2	5.6	5.5	5.3	4.2	98	111	111	123
2180		4	4	4	3	5.7	6.3	6.2	6.2	4.7	5.1	4.9	4.3	93	104	106	119
2181		4	4	4	3	6.4	6.7	6.3	5.6	4.7	5.2	5.0	3.8	113	109	108	117
2183		4	4	4	4	6.6	7.2	6.9	6.8	5.1	5.5	5.4	4.6	107	111	112	125
2227		4	4	4	4	6.7	6.9	7.1	7.0	5.3	5.3	5.3	4.6	106	108	111	116
3655		4	4	5	3	6.4	6.9	6.8	6.3	5.2	5.2	4.7	4.1	94	119	134	127
3656		4	5	4	4	6.4	7.7	7.3	6.9	5.4	5.4	5.0	4.5	104	115	122	121
3658		4	4	4	4	6.3	6.7	6.4	6.2	5.1	5.2	4.8	4.1	98	109	110	121
3685		4	4	4	4	6.5	6.9	7.0	6.9	5.6	5.3	5.2	4.3	103	112	112	123
3695		4	4	4	4	5.9	6.3	6.1	6.3	4.8	5.0	4.9	4.4	101	108	108	110
L1-14	m	4	4	4	4	6.6	6.7	7.0	6.6	5.2	5.3	4.5	4.1	95	95	114	128
LG-03	f	4	4	4	3	6.4	6.8	6.7	6.5	4.8	5.2	5.1	4.3	107	107	111	129
LG-04	f	4	4	4	3	6.2	7.1	6.8	6.1	4.7	5.4	5.0	4.5	110	106	112	111
LG-06	f	4	4	4	4	6.0	6.4	6.6	6.3	4.7	4.7	4.4	4.2	89	97	110	113
Baltic, n = 36																	
20		4	4	4	4	6.4	6.9	6.0	6.2	5.4	5.6	5.0	4.6	94	99	109	119
27	m	4	4	4	3	6.2	6.7	6.7	6.3	5.5	6.0	5.5	3.9	94	110	108	123
34	m	4	4	4	4	6.6	7.0	6.8	6.6	6.3	6.5	5.9	4.5	98	101	103	124
52		4	4	4	4	6.8	7.2	7.4	7.2	4.6	4.7	4.4	4.1	105	109	130	130
59	m	4	4	4	4	6.3	7.1	7.3	6.4	4.7	5.0	4.6	4.2	109	112	128	127
75	f	4	4	4	3	5.7	5.9	6.2	6.2	4.6	4.8	4.6	4.0	96	105	115	124
96	m	4	4	4	3	6.5	6.5	6.7	6.4	5.0	5.3	5.2	4.6	103	112	109	123
104		4	4	4	4	7.3	7.6	7.4	5.9	5.0	5.3	5.2	3.8	102	117	116	132
117	m	4	4	4	3	6.2	6.6	6.5	6.2	5.6	5.7	5.4	4.3	93	94	106	123
125	f	4	4	4	4	7.3	7.8	7.4	6.2	5.2	5.5	5.2	4.5	105	120	118	121
145	m	4	4	4	4	5.9	6.3	6.6	6.7	5.0	5.4	5.1	4.5	95	94	109	111
150	f	4	4	4	4	6.0	6.4	6.2	5.5	5.3	5.3	4.9	4.3	90	98	101	122
151	f	4	5	5	5	6.1	7.2	6.8	6.6	4.8	5.5	5.3	4.3	112	113	118	131
559	m	4	4	4	4	6.2	7.0	6.8	6.5	4.9	5.4	5.1	4.5	103	111	120	124

Population	Sex	Cusp number (n)				Length (mm)				Height (mm)				Angle (degrees)			
ID		P_2	P_3	P_4	M_1	P_2	P_3	P_4	M_1	P_2	P_3	P_4	M_1	P_2	P_3	P_4	M_1
1151		4	4	4	4	6.2	6.6	6.6	6.2	5.2	5.7	5.0	4.5	85	93	107	108
1157		4	4	4	3	7.2	7.6	7.4	6.8								
1164		4	4	4	4	6.3	6.7	6.6	6.2	5.4	5.4	4.9	4.0	96	112	122	128
1166		4	4	4	4	6.1	6.2	6.3	5.7	4.9	5.0	4.8	4.2	99	109	110	118
1167		4	4	4	3	6.6	7.1	7.2	6.7	5.8	6.0	5.5	5.1	92	100	116	121
1170		4	4	4	4	6.8	7.2	6.5	6.3	5.6	5.4	5.1	4.5	90	106	110	110
1172		4	4	4	4	5.9	6.6	6.5	5.8	5.1	5.6	5.3	4.1	97	102	110	120
1178		4	4	4	4	6.3	6.4	6.7	6.6	5.6	5.8	4.9	4.4	96	91	113	115
2264		4	4	4	3	6.5	6.6	5.9	5.7	4.7	4.6	4.2	3.7	112	115	124	140
2265		4	4	4	4	5.9	6.5	6.0	5.7	4.9	5.1	4.6	3.7	101	102	118	129
2267		4	4	4	4	6.7	7.0	6.7	6.1	5.2	5.4	5.1	4.4	100	105	110	117
2275		4	4	4	4	6.6	7.3	6.7	6.6	5.5	5.4	5.0	4.2	102	112	114	123
2276		4	4	4	4	7.2	7.8	7.9	7.4	5.3	5.7	5.2	4.5	106	106	117	120
2277		4	5	5	4	6.5	7.2	6.8	6.9	5.3	5.6	5.2	4.0	110	116	117	126
2279		4	4	4	3	5.6	6.7	6.9	6.1	5.0	5.5	5.2	3.9	102	111	114	122
2280		4	5	4	4	6.4	6.9	6.5	6.4	4.8	5.1	4.8	4.0	111	118	116	128
2281		4	4	4	4	6.8	7.3	7.3	7.1	5.1	5.2	5.4	4.2	101	104	105	125
2285		4	4	5	4	6.4	6.7	7.0	6.8	5.0	5.2	4.7	3.8	98	109	119	128
2289		4	4	5	4	7.0	7.6	7.2	7.0	5.1	4.9	4.4	3.8	108	124	133	140
2306		4	4	4	4	6.7	7.2	6.9	6.5								
2307		4	4	4	4	6.2	7.4	7.1	7.3	4.6	5.2	4.8	4.7	98	103	112	133
2327		4	4	4	3	5.7	6.1	6.4	6.2								
Arctic (Alaska, n = 60)																	
2088	m	4	3	3	3	5.1	5.1	5.0	5.3	4.9	4.9	4.6	4.0	88	97	100	124
3342	m	4	4	4	3	7.0	7.1	6.7	6.5	6.0	6.0	5.4	4.7	100	97	106	117
7114	f	4	4	4	4	6.0	6.9	6.5	6.2								
7118	f	4	4	4	3	5.8	5.9	6.0	5.5	4.6	4.7	4.6	3.7	91	100	106	119
7123	m	4	4	4	4	5.9	6.0	5.9	6.1	5.2	5.1	5.1	4.3	89	98	102	119
11565	m	4	4	4	3	6.2	6.8	6.6	5.9	5.9	6.0	5.8	4.8	90	108	107	116
11566	m	4	4	4	4	5.5	6.2	5.8	6.8	5.2	5.3	5.0	4.6	92	91	95	122
11567		3	4	4	4	6.1	6.5	6.2	6.3	5.0	5.2	4.9	4.5	87	96	103	118
11570	m	4	4	4	4	7.0	7.6	7.6	6.5	5.6	5.5	5.5	4.6	100	113	116	117
11571	f	4	4	4	4	5.4	5.9	5.6	5.5	4.4	4.4	4.1	3.8	100	105	110	118
11572	m	4	4	4	3	5.3	5.8	5.6	5.7	4.5	4.7	4.4	4.0	92	109	112	119
11573	m	4	4	4	4	5.2	6.0	6.1	6.0								
11575	f	3	3	4	3	4.9	5.4	5.4	4.8	4.6	4.5	4.1	3.7	77	94	102	112
11578	f	4	4	4	4	5.9	6.1	5.9	5.9	4.7	4.7	4.5	4.1	94	102	103	119
11582		4	4	4	3	6.1	6.9	6.7	6.4	4.8	5.1	4.7	4.2	94	107	116	123
11585	f	3	4	3	3	5.4	6.2	6.4	6.7	4.9	5.3	5.0	4.5	85	93	112	129
11588	m	4	4	4	4	5.6	6.7	6.4	6.6	5.3	5.6	5.6	4.9	80	94	106	108
11589	f	4	4	4	3	6.2	6.4	6.1	6.2	4.6	5.0	4.6	4.6	101	104	108	121
11590	m	4	4	4	4	7.0	7.8	8.0	7.8	6.1	6.3	5.7	4.8	95	103	116	121
11896	m	4	4	4	3	6.4	7.0	6.2	6.1	5.3	5.5	5.0	4.3	100	108	108	120
11897	f	4	4	4	3	6.0	6.7	6.3	6.1	4.7	5.1	4.7	4.0	105	123	127	131
11899	m	4	4	4	4	6.0	6.4	6.2	6.3	5.1	5.4	5.1	4.7	95	105	110	129
11900	f	3	4	4	4	5.4	6.1	6.0	5.8	5.4	5.5	5.1	4.2	88	101	110	112
15661	m	3	4	4	3	6.1	6.8	6.6	6.1	4.8	5.3	4.8	4.2	95	97	109	112
15677	f	4	4	4	4	6.1	7.0	6.8	6.8	5.1	5.4	5.3	4.6	95	101	111	115
15679	m	4	4	4	3	6.5	7.1	6.9	6.4	4.9	5.5	4.9	4.6	105	101	114	111
15693	m	4	4	4	3	5.6	6.0	6.0	5.5	4.6	5.0	4.5	4.1	86	99	110	127
15699	m	4	4	4	3	6.9	7.2	6.8	6.2	4.6	5.2	4.9	4.4	114	116	117	119
16599		4	5	4	4	6.9	7.1	6.8	6.9	5.9	5.8	5.3	4.6	100	101	107	114
16600		4	4	4	3	6.1	5.8	6.1	5.8								
19060	f	4	4	4	4	5.0	5.3	5.5	5.5	4.4	4.7	4.2	4.1	91	96	106	112
19063	f	4	4	4	4	5.8	6.4	6.1	5.9	4.4	4.5	4.3	3.8	97	112	113	123
19080	f	4	4	4	5	5.5	5.8	5.6	5.5	4.6	4.6	4.3	3.6	98	113	112	124
19084	f	4	4	4	4	6.0	6.1	5.9	5.8								
19094	m	4	4	4	3	5.6	5.7	5.7	5.7	4.8	5.0	4.4	4.0	90	96	103	116
19097	m	4	4	4	4	5.7	6.0	6.0	5.7	4.9	5.0	4.5	4.1	93	99	105	110
19099	m	4	4	4	3	6.3	6.6	6.3	6.0	4.6	4.7	4.4	3.7	98	108	111	112
19118	f	4	4	4	4	6.0	6.6	6.6	5.7	4.8	4.8	4.6	4.1	92	103	108	112
28916	f	4	4	4	4	6.1	6.6	6.7	6.2	4.8	4.7	4.4	4.2	104	119	113	121
28917	f	3	3	3	3	5.9	6.4	6.3	5.9	5.2	5.2	5.0	4.6	82	97	110	113
28918	f	4	4	4	4	5.6	6.2	6.2	6.1	4.7	4.7	4.5	4.2	90	101	110	116
28926	m	4	4	4	3	5.8	6.4	6.8	6.4	5.3	5.4	5.1	4.0	88	100	118	130
30323		4	4	4	3	5.4	5.9	5.8	5.0	4.3	4.5	4.0	3.5	91	100	107	118
33994	f	4	4	4	4	6.8	7.0	7.3	6.9	5.4	5.7	5.4	4.8	100	106	114	114
36249		3	3	3	3	5.4	5.6	5.8	5.7	4.4	4.3	4.1	3.9	94	101	108	114

Population	Sex	Cusp number (n)				Length (mm)				Height (mm)				Angle (degrees)			
ID		P_2	P_3	P_4	M_1	P_2	P_3	P_4	M_1	P_2	P_3	P_4	M_1	P_2	P_3	P_4	M_1
48679	m	4	4	4	4	6.7	7.6	7.1	7.2	5.9	5.7	5.3	5.1	98	116	119	121
63040	m	4	4	4	4	6.3	6.5	6.6	6.5	4.7	5.2	5.1	4.5	113	112	109	119
85496	f	4	4	5	4	5.5	6.2	6.3	5.7	5.5	5.9	5.4	4.6	84	93	106	112
114873	m	4	4	4	3	6.6	7.0	6.7	5.8	4.8	5.1	4.6	4.3	103	112	118	113
114884	f	4	4	4	4	5.7	6.0	6.1	5.5	4.5	4.8	4.5	3.8	97	108	113	121
114891	f	4	4	4	3	5.7	6.7	6.4	6.0								
114929	m	4	4	4	3	6.0	6.7	6.6	5.9	5.1	5.5	4.9	4.2	96	108	110	117
114973	f	4	4	4	3	6.0	6.7	7.0	6.3	5.3	5.8	5.5	4.5	89	97	105	121
116312		4	4	4	3	6.0	6.7	6.4	5.9								
119535	f	4	4	4	4	6.9	6.9	6.6	6.8	5.6	5.6	5.3	4.8	92	99	105	123
119560	f	4	4	4	3	5.3	5.8	5.7	5.4								
126679		4	4	4	3	6.6	6.8	6.9	5.8	5.0	4.9	4.8	4.1	102	106	114	121
126964	m	3	4	4	4	6.9	7.1	6.8	6.7	5.3	5.7	5.3	4.8	101	103	117	123
127073		4	4	3	3	6.3	6.6	6.3	5.5	4.5	4.6	4.3	3.9	102	106	120	115
132082	f	4	4	4	4	6.1	6.3	6.4	5.7	5.6	5.4	4.7	4.2	84	99	113	114
Arctic (Greenland, n = 56)																	
M1218	m	4	4	4	4	6.4	6.9	6.6	6.3	5.2	5.3	4.8	4.0	94	96	109	125
M1219	f	4	4	4	4	5.7	6.1	6.4	6.0	5.3	5.4	4.8	4.0	84	101	120	123
M1220	m	4	4	4	3	5.3	6.3	6.1	6.1	5.0	5.2	4.8	4.3	83	97	103	114
M1223	f	4	4	4	3	5.8	6.2	5.7	5.0	4.2	4.7	4.4	3.5	111	112	114	122
M1224	m	4	4	4	4	5.6	6.3	6.0	5.9	5.1	5.3	4.8	4.6	92	104	108	113
M1225	m	4	4	4	4	5.8	6.6	7.1	6.4	4.6	4.9	4.5	4.2	98	104	121	122
M1226	m	4	4	4	3	5.8	6.0	6.3	5.5	5.0	5.3	5.0	4.3	92	98	109	112
M1230	f	4	4	4	4	6.4	5.9	6.4	6.3	5.0	4.9	4.5	4.0	103	101	108	114
M1232	f	4	4	3	3	5.8	6.3	6.3	6.0	5.3	5.6	5.3	4.5	81	91	98	115
M1233	m	4	4	4	4	6.2	6.5	6.4	6.3	5.2	4.7	5.4	4.2	98	110	107	122
M1234	f	4	4	4	4	5.5	6.1	6.3	5.7	4.4	4.6	4.5	3.8	87	103	106	114
M1236	f	4	4	4	4	5.7	6.2	6.1	5.8	4.6	4.7	4.4	4.1	100	113	131	121
M1238	m	4	4	4	3	7.2	7.6	7.5	6.3	5.8	5.7	5.6	4.4	95	105	116	120
M1241	m	4	4	4	3	5.9	6.4	6.1	6.1	5.2	5.3	5.2	4.1	93	104	103	119
M1244	m	4	4	4	3	5.0	5.8	6.1	5.7	4.5	5.0	4.6	4.4	95	101	110	111
M1245	m	4	4	3	3	6.3	6.6	6.6	6.1	4.6	5.0	4.8	4.2	90	94	108	126
M1246	f	4	4	4	4	6.2	7.1	6.8	6.8	5.1	5.4	4.7	4.2	101	107	116	120
M1248	f	4	4	4	4	5.6	7.1	7.1	6.6	4.5	4.6	4.5	3.9	91	103	113	124
M1249	m	4	4	4	3	6.2	6.5	6.3	6.2	4.9	4.9	4.6	4.2	92	104	114	112
M1255	f	4	4	4	3	6.3	6.8	6.3	5.9								
M1256	f	4	4	4	4	6.1	6.4	6.6	6.6	5.7	6.0	5.3	4.2	88	92	109	125
71		3	3	3	3	5.2	5.9	6.0	6.1	4.3	4.7	4.3	4.3	92	101	121	124
169		4	4	4	3	7.1	7.7	7.3	7.1	5.5	5.5	5.3	4.6	100	101	108	119
171		4	4	4	4	6.4	7.0	6.8	6.6	5.7	5.9	5.5	4.7	92	93	100	109
174		4	4	4	4	6.8	7.7	7.3	6.9	5.6	5.7	5.5	4.8	92	108	110	124
175		4	4	4	4	6.5	7.3	7.2	7.1	5.8	5.9	5.5	5.0	100	105	120	116
186		3	4	3	3	6.9	7.8	7.6	7.2	5.8	5.9	5.5	5.0	100	105	120	116
191		4	4	4	4	6.3	6.9	6.6	6.5	5.1	5.3	5.0	4.3	106	106	112	119
396a		4	4	5	4	6.0	6.7	7.2	7.0	5.0	5.1	5.2	4.5	104	111	112	125
486	f	3	3	3	3	5.0	5.5	5.7	5.6								
487	m	4	4	4	4	5.8	6.0	5.9	6.4	5.0	4.9	4.6	4.1	97	96	108	113
489	f	4	4	4	3	6.6	6.8	7.1	6.5	5.9	5.8	5.6	4.8	95	105	115	118
492	m	4	4	4	4	6.4	6.3	6.2	6.3	4.5	4.3	4.2	4.0	98	110	115	120
494	m	3	4	4	3	5.6	6.5	6.4	6.2	5.3	5.4	5.3	4.8	86	100	100	109
498	f	4	4	4	4	5.5	6.0	6.0	6.0								
577		4	4	4	4	6.9	7.0	7.0	6.9	5.6	5.6	5.3	4.7	102	105	111	122
579		4	4	4	3	5.7	6.1	6.1	5.9	4.7	5.0	4.9	4.5	98	107	107	106
588		4	4	4	3	7.2	7.3	7.0	6.3	5.9	5.5	5.4	4.5	95	106	107	120
590		4	4	4	3	6.8	7.6	6.9	6.6	5.2	5.6	5.0	4.2	98	104	109	112
594	m	4	4	4	4	7.0	7.5	7.4	7.4	5.7	5.6	5.4	4.8	97	111	112	123
596	m	4	4	5	4	6.4	6.9	7.0	6.2	5.0	5.1	4.8	4.2	104	108	117	118
597	f	4	4	3	3	6.2	6.6	6.2	5.5	4.4	5.1	4.7	3.7	108	104	113	129
598	m	4	4	4	3	6.0	7.1	6.7	6.4	5.5	5.7	5.3	4.3	89	103	107	121
600		5	5	5	5	6.8	7.6	7.4	6.6	5.3	5.7	5.4	4.3	104	110	114	121
603	m	4	4	4	4	5.5	6.2	6.0	6.1	4.1	4.4	4.1	3.9	98	108	112	113
604		4	4	4	3	6.1	6.6	6.4	6.1	4.6	5.0	5.0	4.8	100	121	109	110
605		4	4	4	3	5.5	5.9	6.1	6.2	5.5	5.6	5.1	4.2	85	93	107	108
606	m	4	4	4	4	5.5	6.2	6.5	6.0								
608	m	4	4	4	4	7.2	7.5	7.6	6.7	6.1	6.0	5.7	4.5	100	111	112	116
710	f	4	4	4	3	6.7	7.6	7.0	6.6								
719	f	4	4	4	3	6.0	6.7	6.4	5.9	5.3	5.7	5.3	4.4	88	93	95	113
798	m	4	4	4	3	6.1	6.6	6.4	5.8	4.9	4.9	4.6	4.2	86	98	114	113

Population	Sex	Cusp number (n)				Length (mm)				Height (mm)				Angle (degrees)			
ID		P_2	P_3	P_4	M_1	P_2	P_3	P_4	M_1	P_2	P_3	P_4	M_1	P_2	P_3	P_4	M_1
799	f	4	4	4	4	6.8	7.4	6.8	6.5	5.4	5.7	5.0	4.8	93	96	106	110
1185		4	4	4	4	6.3	7.0	6.7	6.4	5.2	4.8	4.3	3.6	103	113	118	134
1186		3	4	4	3	6.5	7.1	7.3	6.3	5.4	5.4	5.1	4.3	91	101	111	120
1187		4	4	4	3	5.1	5.8	5.4	4.8	4.2	4.6	4.2	3.5	95	100	107	119
Arctic (Kara Sea, n = 38)																	
M30062	m	4	4	4	4	6.6	7.1	7.0	6.5	5.4	5.5	4.9	4.5	101	107	121	118
M30063	f	4	4	4	4	6.9	6.9	6.8	6.0	5.3	5.7	4.9	4.2	95	96	110	110
M30065	f	4	4	4	4	6.4	6.0	6.3	5.7								
M30067	f	4	4	4	4	5.2	5.9	5.8	5.7								
M30070	m	4	4	5	4	6.0	6.6	6.8	6.4								
M30071	m	4	4	4	3	5.0	5.9	5.6	5.5	3.9	4.4	4.2	4.0	88	92	95	98
M30072	m	4	4	4	4	6.6	7.0	6.4	6.6	5.0	4.9	4.6	4.1	94	106	103	124
M30073	m	4	4	3	3	6.6	7.3	6.6	6.5	5.4	5.7	5.2	4.7	90	102	109	119
M30075	m	4	4	4	3	5.6	5.6	5.7	5.9	4.8	5.0	4.8	4.4	90	95	99	108
M30076	m	4	4	4	3	6.2	6.9	6.8	6.4	5.4	5.6	5.1	4.6	85	101	106	113
M30077	m	4	4	4	4	5.6	6.8	6.1	6.1								
M30078	m	4	4	4	4	6.0	6.5	6.4	6.3	5.4	5.8	5.6	4.8	86	99	104	103
M30079	f	4	4	4	3	6.5	7.3	6.6	5.8								
M30080	f	4	4	4	4	5.4	6.2	6.0	5.0								
M30081	m	4	4	4	3	6.3	6.5	6.7	5.9	5.1	5.4	5.4	4.8	96	102	106	116
M30085	m	5	5	5	5	7.1	7.6	7.7	6.8	5.5	5.8	5.3	4.5	96	109	114	116
M30086	m	4	4	4	4	6.2	7.3	7.3	6.7								
M30087	m	3	3	3	4	5.5	5.9	6.2	5.9								
M30088	m	4	4	4	3	6.2	6.4	6.4	6.1	4.8	5.1	4.7	4.2	91	93	106	115
M30089	m	4	5	5	5	5.9	6.6	6.8	6.6								
M30090	m	4	4	4	4	5.6	6.4	6.3	6.1	5.3	5.4	5.0	3.9	72	97	107	116
M30091	m	4	4	5	4	5.6	6.8	6.3	5.6	4.9	5.4	4.9	4.1	90	105	112	115
M30092	m	4	4	4	4	6.6	6.7	6.6	6.5	5.1	5.4	4.9	4.3	91	102	112	115
M30093	m	4	4	5	3	6.3	6.3	6.3	5.6	5.0	4.9	4.4	4.3	93	98	105	112
M30094	m	4	4	4	3	6.4	6.8	6.8	6.3								
M30095	m	4	4	4	3	6.7	7.1	7.2	6.0	5.0	5.5	4.9	4.3	92	100	112	113
M30096	m	4	4	4	3	6.2	7.5	7.4	6.6								
M30097	f	4	4	4	3	5.8	6.1	5.9	5.6								
M30098	m	3	4	3	3	5.8	6.3	6.1	6.0	4.3	4.6	4.1	3.9	96	105	116	124
M30099	m	4	4	4	4	6.2	6.5	6.4	6.0	5.0	5.1	4.8	4.6	87	94	106	104
M30100	m	4	4	4	3	6.4	6.8	6.6	5.9	5.6	5.3	5.2	4.4	88	97	102	116
M30101	m	4	4	4	4	6.5	6.9	6.7	6.4	4.8	4.9	4.5	4.2	106	124	130	128
M30102	m	4	4	4	3	6.7	6.9	6.7	6.0	4.8	5.2	4.9	4.3	105	108	112	123
M30103	m	4	4	4	4	6.2	7.5	7.3	6.4	5.3	5.6	5.2	4.9	96	104	112	113
M30104	m	3	4	3	3	5.7	6.3	6.3	6.1								
M30105	m	4	4	4	4	6.1	6.4	6.4	6.5	3.8	4.3	4.1	4.0	96	96	106	125
M30106	m	4	4	4	4	5.7	6.2	6.1	5.8	4.4	4.4	4.2	4.1	88	107	119	111
M30107	m	4	4	4	4	5.7	5.9	5.8	6.2	4.7	5.0	4.7	4.4	89	90	92	103
Okhotsk, n = 19																	
M12846	m	4	4	4	4	6.6	6.9	6.6	6.5	4.9	5.5	5.1	4.2	99	105	105	114
M12954		4	4	4	3	5.5	6.2	6.2	5.9	4.7	4.8	4.6	4.1	91	103	107	111
M28991	f	3	4	3	3	5.3	5.3	5.4	5.4								
M28993	m	4	4	4	4	5.6	6.0	6.1	6.2	4.5	4.9	4.8	4.3	115	111	109	127
M29022	f	3	4	4	3	4.9	5.8	6.3	5.5	3.9	4.3	4.2	3.6	102	115	125	124
M29030	m	4	4	4	4	6.2	6.4	6.4	6.2	4.6	4.8	4.8	4.1	111	112	117	115
M29032	f	4	4	4	4	5.3	6.4	6.2	6.1								
M29034	m	4	4	4	3	5.7	5.9	5.6	5.7	4.4	4.7	4.4	4.0	95	115	125	122
M29060		4	4	4	3	4.9	5.7	5.8	5.5	4.2	4.5	4.4	3.9	84	97	104	114
M29061		4	4	4	4	6.0	6.5	6.5	6.5	4.8	4.8	4.4	4.0	105	115	121	124
M29062	f	3	4	4	3	5.3	5.9	6.0	5.2								
M42632	f	4	4	3	3	5.2	5.7	5.4	5.0								
M42634	m	4	4	4	3	5.4	6.2	6.0	5.8								
M42635	f	3	4	4	3	6.0	6.9	6.6	6.2	4.9	4.8	4.4	3.6	88	111	119	130
M42636	f	4	4	4	3	5.6	6.3	6.1	5.3	4.5	4.6	4.5	3.7	97	104	111	112
M42637	m	4	4	4	4	5.5	6.3	6.2	5.8	3.7	3.9	3.6	3.2	105	113	127	131
M46970	m	4	4	4	3	5.4	5.8	5.5	5.6	4.7	4.3	4.0	3.8	89	107	115	115
M46971	m	3	4	4	3	6.1	6.5	5.9	4.9	4.6	4.5	4.0	3.8	92	106	124	109
M72197	m	4	4	5	5	6.4	6.5	6.8	6.6								

The full uri specimen IDs for the Ladoga and Baltic specimens are of the form https://id.luomus.fi/KS.KN1160 etc.

Table S4. Summary statistics of tooth measurements for P_2 to M_1. Data are from the right side. Arctic sample has specimens from Alaska, Greenland, and Kara Sea (*SI Appendix*, Table S3).

	Cusp number			Length			Height			Top-cusp angle		
	Mean	Range	SD	Mean	Range	SD	Mean	Range	SD	Mean	Range	SD
Saimaa												
P_2	3.7	3-4	0.45	5.8	4.5-7.0	0.54	5.3	4.6-6.0	0.34	91.1	77-102	5.70
P_3	4.0	3-4	0.20	6.5	5.4-7.5	0.41	5.4	4.5-6.1	0.35	99.8	83-112	5.61
P_4	4.0	3-4	0.14	6.4	5.0-7.1	0.43	4.9	3.8-5.4	0.33	111.8	95-126	5.91
M_1	3.2	3-4	0.40	5.3	3.9-7.2	0.60	4.1	3.4-4.8	0.27	111.8	95-123	6.22
Ladoga												
P_2	4.0	3-4	0.12	6.2	5.1-7.3	0.44	5.1	4.5-5.9	0.34	99.2	80-117	7.33
P_3	4.0	4-5	0.21	6.7	5,2-7.7	0.45	5.3	4.7-5.9	0.29	106.7	88-119	6.66
P_4	4.1	4-5	0.24	6.6	5.4-7.9	0.47	5.0	4.3-5.6	0.30	112.3	96-134	5.88
M_1	3.6	3-5	0.58	6.2	5.0-7.4	0.46	4.2	3.5-4.8	0.33	120.7	110-134	5.97
Baltic												
P_2	4.0	4-4	0.00	6.4	5.6-7.3	0.45	5.2	4.6-6.3	0.38	100.1	85-112	6.79
P_3	4.1	4-5	0.28	6.9	5.9-7.8	0.48	5.4	4.6-6.5	0.39	107.1	91-124	8.14
P_4	4.1	4-5	0.32	6.8	5.9-7.9	0.46	5.0	4.2-5.9	0.36	114.5	101-133	7.45
M_1	3.8	3-5	0.48	6.4	5.5-7.4	0.46	4.2	3.7-5.1	0.33	123.8	108-140	7.29
Arctic												
P_2	3.9	3-5	0.33	6.1	4.9-7.2	0.54	5.0	3.8-6.1	0.48	94.4	72-114	7.16
P_3	4.0	3-5	0.27	6.6	5.1-7.8	0.57	5.2	4.3-6.3	0.45	102.9	90-124	6.80
P_4	4.0	3-5	0.39	6.5	5.0-8.0	0.53	4.9	4.0-5.8	0.44	110.1	92-131	6.59
M_1	3.5	3-5	0.55	6.1	4.8-7.8	0.50	4.3	3.5-5.1	0.35	117.4	98-134	6.15
Okhotsk												
P_2	3.7	3-4	0.45	5.6	4.9-6.6	0.48	4.5	3.7-4.9	0.37	97.9	84-115	9.33
P_3	4.0	4-4	0.00	6.2	5.3-6.9	0.42	4.6	3.9-5.5	0.38	108.8	97-115	5.59
P_4	3.9	3-5	0.40	6.1	5.4-6.8	0.41	4.4	3.6-5.1	0.39	116.1	104-127	8.18
M_1	3.4	3-5	0.61	5.8	4.9-6.6	0.51	3.9	3.2-4.3	0.30	119.1	109-131	7.54

Table S5. Permutation tests between Saimaa and other samples for tooth length, tooth height, height/length ratio (H/L), and top-cusp angle. *P*-values are one-tailed and except for the ones marked with asterisk, denote lower values for Saimaa lengths and top-cusp angles, and higher values for heights and height/length ratios. Tests are between group means using 10 000 permutations, *P*-values below 0.05 are marked in bold.

Length	Ladoga	Baltic	Arctic	Okhotsk	Angle	Ladoga	Baltic	Arctic	Okhotsk
P_2	**0.0000**	**0.0000**	**0.0061**	0.0623*	P_2	**0.0000**	**0.0000**	**0.0020**	**0.0051**
P_3	**0.0202**	**0.0001**	0.2003	**0.0019***	P_3	**0.0000**	**0.0001**	**0.0031**	**0.0000**
P_4	**0.0095**	**0.0002**	0.3261	**0.0018***	P_4	0.2596	0.0641	0.0671*	**0.0260**
M_1	**0.0000**	**0.0000**	**0.0000**	**0.0011**	M_1	**0.0000**	**0.0000**	**0.0000**	**0.0006**

Height	Ladoga	Baltic	Arctic	Okhotsk	H/L	Ladoga	Baltic	Arctic	Okhotsk
P_2	**0.0145**	0.1325	**0.0026**	**0.0000**	P_2	**0.0000**	**0.0000**	**0.0000**	**0.0000**
P_3	**0.0126**	0.3291	**0.0020**	**0.0000**	P_3	**0.0001**	**0.0008**	**0.0000**	**0.0001**
P_4	0.1663*	0.1151*	0.2045	**0.0000**	P_4	0.0571	0.1181	0.0513	**0.0035**
M_1	0.0559*	0.0772*	**0.0012***	**0.0027**	M_1	**0.0000**	**0.0000**	**0.0000**	**0.0000**

Table S6. Shape profiles of the tongues. Tongues were divided into ten-percent bins from the tip to the end of the posterior body. *Lobodon* and *Hydrunga* are measured from (6). Saimaa and Baltic material are from Metsähallitus (the Finnish Forest Administration).

	Relative tongue width (%) from the tip of the tongue							
Specimen (kg)	10%	20%	30%	40%	50%	60%	70%	80%
Saimaa 2698 (38)	66	76	83	92	97	100	100	95
Saimaa 2664 (41)	64	70	83	90	100	97	90	90
Saimaa 2732 (36)	64	75	80	83	100	98	98	96
Saimaa 2728 (25)	66	77	88	98	100	99	95	78
Saimaa 2704 (35)	58	68	79	89	100	98	97	89
Saimaa 2747 (18)	64	70	79	90	97	100	98	90
Saimaa 2734 (24)	71	73	77	84	92	100	98	92
Baltic 459 (46)	53	61	68	75	83	93	100	94
Hydrurga	49	56	63	70	77	89	100	99
Lobodon	56	68	76	87	96	100	94	87

Table S7. Crown-relief and cusp index (7) measurements. Values plotted in Fig. 5*C* are mean reliefs of the four postcanines. *P*-values between means of Saimaa and the other three samples are from 0.001 to 0.008 for relief and 0.001 to 0.025 for cusp index (one-tailed Mann-Whitney *U* test).

		Crown relief				Cusp index			
		P_2	P_3	P_4	M_1	P_2	P_3	P_4	M_1
Saimaa									
	UEF 759	3.10	3.49	3.06	2.82	1.02	1.07	1.02	0.99
	UEF 821	2.78	2.79	2.75	2.87	0.96	1.00	1.04	0.97
	UEF 1193	2.80	2.78	2.85	2.73	1.08	1.06	1.06	1.00
	UEF 2379	3.45	3.44	3.37	3.07	1.10	1.13	1.17	1.01
	UEF 2386	2.87	3.10	3.13	2.65	0.98	1.04	1.05	0.98
	KS.KN5655*	2.77	2.62	2.80	2.51	1.00	0.99	1.05	0.99
	KS.KN5688b*	3.15	3.09	2.77	2.76	0.98	1.02	1.02	0.92
Baltic									
	KS.KN483	3.46	3.36	3.46	2.80	0.99	1.03	1.04	0.99
	KS.KN1164	3.56	3.25	3.20	2.71	1.02	1.07	1.07	1.03
	KS.KN2267	3.89	3.59	3.29	3.38	1.08	1.07	1.04	1.01
	KS.KN2275	3.51	3.27	3.12	2.90	1.08	1.09	1.05	1.00
	KS.KN2280	3.48	3.61	3.28	2.91	1.04	1.06	1.04	0.99
	KS.KN2281	3.31	3.23	3.35	2.89	1.10	1.14	1.06	1.05
	KS.KN47154	3.55	3.42	3.58	3.18	1.09	1.11	1.09	1.07
	KS.KN47190	3.04	2.96	2.97	3.50	1.06	1.08	1.07	0.99
Hydrurga									
	NMV 7384	3.37	3.14	3.44	3.00	1.15	1.12	1.18	1.13
	NMV 13866	3.50	3.21	3.52	2.75	1.11	1.11	1.13	1.07
	NMV 23589	3.13	3.42	3.57	2.98	1.10	1.10	1.15	1.12
	NMV 30849	3.29	3.44	3.32	3.14	1.16	1.14	1.19	1.14
	NMV 31561	3.48	3.43	3.28	-	1.11	1.14	1.13	-
	NRM 620682	3.27	3.56	3.65	3.39	1.07	1.12	1.14	1.16
Lobodon									
	NMV 7385	3.21	3.52	3.47	3.50	1.10	1.14	1.12	1.15
	NMV 7392	3.60	3.64	3.96	4.12	1.11	1.19	1.24	1.30
	NMV 7392	3.74	3.81	4.27	4.24	1.08	1.19	1.22	1.29
	NMV 7396	-	3.33	3.80	3.77	-	1.11	1.16	1.25
	NRM 895081	3.26	3.29	3.27	3.94	1.10	1.14	1.15	1.25
	NRM 895082	3.17	3.61	3.86	3.83	1.06	1.19	1.23	1.31

* The full uri specimen IDs for the FMNH specimens are of the form https://id.luomus.fi/KS.KN5655

References

1. A. Löytynoja *et al.*, Fragmented habitat compensates for the adverse effects of genetic bottleneck. *Curr Biol* **33**, 1009-1018 e1007 (2023).
2. A. Rosing-Asvid *et al.*, An evolutionarily distinct ringed seal in the Ilulissat Icefjord. *Mol Ecol* 10.1111/mec.17163 (2023).
3. J. Y. Park *et al.*, Deciphering the evolutionary signatures of pinnipeds using novel genome sequences: The first genomes of Phoca largha, Callorhinus ursinus, and Eumetopias jubatus. *Sci Rep* **8**, 16877 (2018).
4. O. Dudchenko *et al.*, De novo assembly of the Aedes aegypti genome using Hi-C yields chromosome-length scaffolds. *Science* **356**, 92-95 (2017).
5. S. Y. Xu, X. Y. Xie, R. R. Zhao, J. S. Tian, Z. C. Lu, Genomic comparison of male and female spotted seals in the Liaodong Gulf, China. *Reg Stud Mar Sci* **76** (2024).
6. C. M. Loza *et al.*, Description and distribution of the mechanical papillae of the lingual surface of Antarctic seals species (Phocidae: Carnivora) and their relationship with diet and dental morphology. *Polar Biol* **46**, 235-251 (2023).
7. U. Ishihara, N. Miyazaki, D. J. Yurkowski, Y. Y. Watanabe, Multi-cusped postcanine teeth are associated with zooplankton feeding in phocid seals. *Marine Ecology Progress Series* **729**, 233-245 (2024).
